# Morphological and Molecular Distinctions of Parallel Processing Streams Reveal Two Koniocellular Pathways in the Tree Shrew DLGN

**DOI:** 10.1523/ENEURO.0522-24.2025

**Published:** 2025-07-03

**Authors:** Francesca Sciaccotta, Arda Kipcak, Alev Erisir

**Affiliations:** Department of Psychology, University of Virginia, Charlottesville, Virginia 22903

## Abstract

In the mammalian visual system, three functionally distinct parallel processing streams extend from the retina to the visual thalamus and then to the visual cortex: magnocellular (M), parvocellular (P), and koniocellular (K). Tree shrews (*Tupaia belangeri*), a preprimate species, provide an advantageous model to study the K pathway in isolation because, while M and P pathways remain mixed in Lamina 1 (L1), L2, L4, and L5 of the lateral geniculate nucleus (LGN), L3 and L6 receive strictly K-input from the contralateral eye. Additionally, K-input laminae selectively receive glutamatergic axons from the superior colliculus. To reveal how cellular and synaptic properties of K geniculate laminae may differ from M/P laminae and how tectal input may shape the K relay to the cortex, we studied the morphology and connectivity of retinal and tectal terminals in pathway-specific laminae. While confirming that K laminae relay cells contain calbindin, we also found its expression in GABAergic cells across all laminae. No cell-type or lamina specificity was observed for parvalbumin. Ultrastructurally, retinal terminals are morphologically distinct in M/P versus K laminae. Tectogeniculate axons in L3 and L6 resemble retinal terminals in their morphology and synaptic targets, while corticogeniculate terminals are sparse in L6. VGluT2, the molecular marker for large-sized driver terminals, is expressed prominently in one of the three tectal cell types that project to LGN. Morphological differences in synaptic circuitry between L3 and L6 provide further evidence that two geniculate K laminae are differentially innervated to relay distinct sets of information to the cortex.

## Graphical abstract

**Figure:**
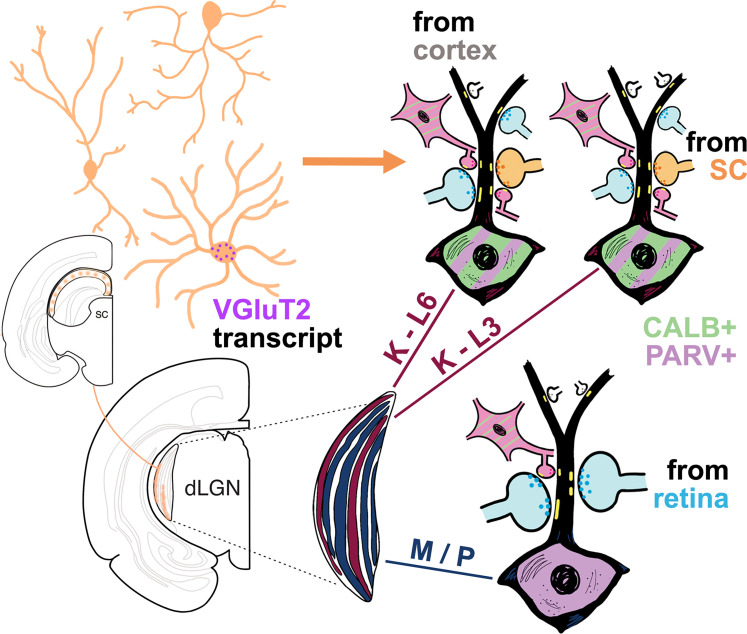
Visual Abstract

## Significance Statement

The current study provides new insights into the unique organization and functional roles of koniocellular (K) pathways in visual processing. Using the tree shrew's unique lamina organization in the lateral geniculate nucleus, where magnocellular (M)/parvocellular (P) and K pathways remain segregated, we reveal structural and neurochemical differences between these processing streams. Our findings are consistent with the idea that two geniculate K laminae are distinct and their circuitry may support adaptive roles, such as motion detection and visual processing and potentially maintaining visual function in conditions like blindsight. This research underscores the K pathway's heterogeneity and distinctions from M/P pathways and its importance in visual processing and adaptability.

## Introduction

Anatomical and physiological studies have identified three main types of relay cells that extend from the retina to the cortex in most mammalian species examined: magnocellular (M), parvocellular (P), and koniocellular (K) in primates (Kaas et al., 1978; Irvin et al., 1986; Kaplan, 2003, 2014) and X, Y, and W cells in other mammals (Enroth-Cugell and Robson, 1966; Friedlander et al., 1981; Holdefer and Norton, 1995; Van Hooser et al., 2003). In most species, the lateral geniculate nucleus (LGN) is organized into laminae which are devoted to one or more parallel processing streams (Campbell et al., 1967; Kaas et al., 1978; Holdefer and Norton, 1995; Van Horn et al., 2000; Kaplan, 2003, 2014; Kerschensteiner and Guido, 2017). The geniculate neurons are located in pathway-specific laminae and may show distinct morphological and response property characteristics that reflect the RGC origin of the primary, driving input, indicating functionally distinct parallel pathways.

In primates, tree shrews, and some carnivores, parallel processing streams originating from the retina remain segregated in the LGN, although segregation patterns of these parallel pathways vary across species. In the preprimate species, tree shrew (*Tupaia belangeri*), Lamina (L)1, L2, L4, and L5 all receive mixed projections comprised of both M and P RGC axons, while L3 and L6 are solely comprised of K axons (Conway and Schiller, 1983; Conley et al., 1984; Holdefer and Norton, 1995). Thus, M and P pathways overlap in geniculate laminae, while the K pathway is segregated, which highlights the tree shrew species as a suitable model to study the K pathway in isolation.

Tree shrews also serve as an advantageous animal model because of their LGN laminae organization and parallel pathway segregation. The organization of the tree shrew dLGN parallel pathways contains three pairs of layers (Holdefer and Norton, 1995): L1 and L2, L4 and L5, and L3 and L6. L1 and L2 form a pair that receives ipsilateral and contralateral projections, respectively, from medium- to large-sized RGCs and display ON center receptive field properties. L4 and L5 form a pair that receives contralateral and ipsilateral projections, respectively, from medium- to large-sized RGCs and display OFF center receptive field properties. The only layers that receive input from the superior colliculus (SC), L3 and L6 (Diamond et al., 1991), form a pair that receives contralateral projections from small-sized RGCs and display W-like (K) and ON–OFF center receptive field organization (Conway and Schiller, 1983; Holdefer and Norton, 1995). These patterns reveal that the K pathway is a distinct part of the afferent visual stream in tree shrews with apparent differences from M/P parallel pathways.

In this study, we asked if the morphological, molecular, and synaptic properties of neurons and axonal inputs in K-specific lamina of the tree shrew LGN are different from those in non-K lamina. While three major classes of RGCs have been identified morphologically based on their axon diameter, soma area, and dendritic field (DeBruyn, 1983; Drenhaus et al., 1997; Lu and Petry, 2003), there is no direct demonstration showing that these phenotypic features are maintained in the LGN, although clustering analyses of retinal terminals in mouse LGN suggests that different RGC types may have unique bouton morphology and size (Maher et al., 2023). We utilized immunohistochemical and tract-tracing methods to study the cellular and morphological properties of the parallel processing streams in geniculate lamina. Overall, our findings suggest that there are chemical, synaptic, and morphological distinctions of parallel pathways in the tree shrew dLGN, with evidence that suggests the existence of two subpathways of the K processing stream.

## Materials and Methods

### Animals

Data for this study were collected from the brains of 10 adult tree shrews (*Tupaia belangeri*) of both sexes. All procedures were approved by the University of Virginia Institutional Animal Care and Use Committee and were conducted in accordance with the National Institutes of Health guidelines.

### Tract-tracer injections

In order to label tectogeniculate (TG) projections via anterograde transport, adult tree shrews were anesthetized with 2–5% isoflurane and placed on a stereotaxic apparatus. An incision was made along the scalp, and a craniotomy was performed above the SC. The stereotaxic coordinates used for SC injections were as follows: AP, −6.0 to 7.5; ML, 2.0–2.5; and DV, 3.5–4.0 mm (Zhou and Ni, 2016). A NanoFil 10 μl syringe [World Precision Instruments (WPI)] containing 300 nl of a 5% solution of biotinylated dextran amine (BDA-10,000; Invitrogen) in saline or 400 nl of undiluted AAV-CAG-tdTomato (Table 1) was lowered into the SC and infused at a rate of 1 nl/s using Legato 130 Nanosystem (KD Scientific). The needle was left at the target for an additional 5 min at the end of the injection cycle to minimize backflow before it was retracted. The craniotomy was sealed with bone wax, the scalp was sutured, and the animals were placed on a heating pad until mobile. After the surgery, animals were monitored for 3 d to ensure proper wound healing and were observed for any behaviors indicative of pain or discomfort.

**Table 1.** Antibodies, reagents, and tracers used in this study

Name	Info	Dilution
AAVrg-CAG-GFP	Addgene; #37825	2 × 10^13^ vg/ml
Alexa Fluor 488 anti-mouse IgG	Thermo Fisher Scientific; #A21202 (donkey, polyclonal)	1:250
Alexa Fluor 488 anti-rabbit IgG	Thermo Fisher Scientific; #A21206 (rabbit, polyclonal)	1:250
Anti-CALB-D-28K	Sigma-Aldrich; #​C9848 (mouse, monoclonal)	1:1,000
Anti-GABA	Sigma-Aldrich; #A2052 (rabbit, polyclonal)	1:250
Anti-PARV	Sigma-Aldrich; #P3088 (mouse, monoclonal)	1:200
Anti-vesicular glutamate transporter 2 (VGluT2)	EMD Millipore; #AB2251 (guinea pig, polyclonal)	1:2,500
Biotin anti-guinea pig IgG	Vector; #A32731 (goat, polyclonal)	1:50
Biotin dextran amine (BDA-10,000)	Invitrogen; #D1956	5 mg/ml
CTB 555	Thermo Fisher Scientific; #C34776	5 mg/ml
Cy5 AffiniPure anti-guinea pig IgG	Jackson ImmunoResearch Laboratories; #706-175-148 (donkey, polyclonal)	1:250
NT Nissl Stain	Thermo Fisher Scientific; #N21479	1:250
Streptavidin Alexa Fluor 647	Abcam; #AB272190 (Monovalent)	1:250
TSA Vivid Fluorophore 650	Advanced Cell Diagnostics; #323273	1:1,000

In order to label SC cells that project to LGN via retrograde tracers, adult tree shrews were anesthetized with 2–5% isoflurane and placed on a stereotaxic apparatus. An incision was made along the scalp, and a craniotomy was performed above the LGN. The stereotaxic coordinates used for LGN injections were as follows: AP, −3.8 to 4.8; ML, 4.6–4.8; and DV, 5.5–6.5 mm (Zhou and Ni, 2016). A Nanofil 10 μl syringe (WPI) containing 300 nl of undiluted AAVrg-EGFP (Table 1) was lowered into the LGN and infused at a rate of 1 nl/s using Legato 130 Nanosystem (KD Scientific).

For the eye injections, the animals were anesthetized with isoflurane (SomnoFlo; Kent Scientific). A Hamilton 25 μl syringe (Hamilton Company, Reno, NV; #80408) was filled with cholera toxin B (CTB; 5 mg/ml; Thermo Fisher Scientific) conjugated to Alexa Fluor 555 or BDA (5%). The needle was inserted at the nictitating membrane close to the lateral canthus to access the posterior chamber, and 5–10 μl of the tracer was injected.

### Tissue preparation

After a minimum of 21 d required for tracer transport, the animals were deeply anesthetized with an overdose of Euthasol (excess of 0.25 ml/kg, i.p.) and transcardially perfused with Tyrode's solution (in mm: 137 NaCl, 2 KCl, 0.9 CaCl_2_, 1.2 MgCl_2_, 11.9 NaHCO_3_, 0.4 NaH_2_PO_4_, 5.5 glucose; 281 mOsm), pH 7.4, for 1–2 min, followed by 300 ml of a fixative solution containing 4% paraformaldehyde (PFA) and 0.0 (for fluorescent labeling experiments) or 0.5% (for EM experiments) glutaraldehyde in 0.1 M phosphate buffer (PB), pH 7.4, at 30–35°C. Brains were extracted and postfixed overnight in 4% PFA at 4°C. Subsequently, brains were blocked and sectioned coronally at 50–60 μm on a Leica VT 1000 S vibratome (Leica Biosystems). Sections were then rinsed in 1% sodium borohydride and stored in 0.05% sodium azide (NaN_3_) in 0.01 M PBS at 4°C prior to immunohistochemistry.

### Immunohistochemistry

Table 1 lists the primary and secondary antibodies and the dilutions used in the current study. Sections for confocal imaging were placed in the primary antibody diluted in PBS solution containing 1% bovine serum albumin (BSA), 0.3% Triton X-100, and 0.05% NaN_3_ for 24–48 h at room temperature (RT) on a shaker. To terminate the incubation, we rinsed the sections in 0.01 M PBS and incubated in a secondary antibody conjugated to Alexa fluorophores for 2 h. Sections were then rinsed, mounted onto subbed slides, and coverslipped with VECTASHIELD Mounting Media.

For electron microscopy, sections were preincubated in 0.01 M PBS containing 1% BSA and 0.1% Triton X-100 for 30 min. Sections were then transferred into a primary antibody diluted in 0.01 M PBS containing 1% BSA and 0.05% NaN_3_ for 72 h on a shaker. To terminate the incubation, we rinsed the sections in 0.01 M PBS and incubated them in a secondary antibody conjugated to biotin for 2 h. This was followed by an incubation in the avidin–biotin complex (Vector Laboratories) solution for 2 h. Sections were then rinsed in 0.01 M PBS, and the labeling was visualized in a solution of 0.02% hydrogen peroxide (H_2_O_2_) and 0.05% diaminobenzidine (DAB) for 2–7 min.

### RNA fluorescence in situ hybridization (FISH)

Free floating sections containing the SC were dissected out and incubated in hydrogen peroxide for 10 min in a well plate. After incubation, slices were washed three times for 10 min in PBS to remove bubbles, mounted onto a SuperFrost Plus slide (Thermo Fisher Scientific; catalog #12-550-15) in a square configuration, and left to air-dry. The slide was then baked in an oven at 40°C for 30 min, followed by incubation in 4% PFA at 4°C for 15 min and dehydration in 50, 70, and 100% ethanol at RT for 5 min each. The slide was then air-dried for 5 min at RT, boiled in distilled water at 99°C for 10 s, incubated in target retrieval buffer at 99°C for 5 min, rinsed in distilled water for 15 s at RT, and lastly dehydrated in 100% ethanol for 3 min at RT. The slide was air-dried and left overnight in a closed container at RT.

The following day, RNAscope Multiplex v2 (Advanced Cell Diagnostics, catalog #323270) was used to label specific mRNA transcripts. The slide-mounted sections of the SC were outlined with a hydrophobic barrier, rinsed with distilled water, digested with Protease IV at 40°C for 30 min, and incubated in a *Slc17a6* probe for VGluT2 (vesicular glutamate transporter type 2; catalog #1269961-C1) at 40°C for 2 h. The slide was then treated with amplification reagents (AMP 1–3) at 40°C for 15–30 min each. Following this, HRP-C1 and TSA Vivid Dye 650 were applied for 15–30 min at 40°C to bind to AMP 3 and activate fluorescence. The slide was then washed in a series of 0.1 M PBS and distilled water rinses and left to air-dry before being cover slipped with Aqua-Poly/Mount mounting medium (catalog #18606).

### Embedding for electron microscopy

Using routine protocols, sections were treated with 1% osmium tetroxide (OsO_4_) in 0.1 M PB for 1 h. Sections were then treated with filtered 4% uranyl acetate in 70% alcohol for 1 h, followed by dehydration in acetone and treatment with a 1:1 acetone/resin mixture overnight. The following day, sections were transferred to full resin [EMBED 812; Electron Microscopy Sciences (EMS)] and left overnight. Sections were then flat embedded between two Aclar sheets (EMS) and cured in a 60°C oven overnight. Sections of LGN to be used for EM were identified from flat embed sections and photographed with a light microscope. The sections containing the tree shrew LGN were excised and placed in BEEM capsules (EMS). The capsules were filled with resin and cured at 60°C for 24–48 h or until polymerized. The region of interest including laminar borders and the other landmarks such as capillaries and myelinated axon bundles was traced with a camera lucida, and the capsule embedded tissue was trimmed down to a 1 × 2 mm trapezoid containing the entirety of LGN laminae and the optic tract. Ultrathin sections of ∼50–80 nm thickness were collected on 400 mesh copper grids (Ted Pella) using an ultramicrotome (Ultracut UCT7; Leica Microsystems). The geniculate laminae in ultrathin sections were identified with the help of trapezoid orientation over capsule-embed and the tissue landmarks including optic tract border.

### Imaging, statistical modeling, and analyses

For confocal microscopy, 60-μm-thick coronal sections were mounted on slides, dried, and coverslipped with Aqua-Poly/Mount mounting medium (catalog #18606). Images were subsequently collected using Leica Stellaris 5 laser scanning confocal microscope using HC PL APO 40×/1.3 NA and HC PL APO 63×/1.4 NA with a maximum voxel resolution of 0.06 × 0.06 × 0.3 µm for highest-resolution images.

For EM images, ultrathin sections on copper grids were examined on a JEOL1010 electron microscope equipped with a 16 megapixel CCD camera (SIA). For quantitative morphology and immunolabeled terminal analysis, overlapping images were captured within laminar borders at the lipid membrane bilayer resolution. Images for quantitative and immunolabeled terminal analysis were taken at 8,000–15,000× magnification, yielding a pixel size resolution of 0.8–1.7 nm. ImageJ (NIH) and the Microscope Measurement Tools plugin were used to quantify the terminal area and synapse length for both unlabeled and labeled terminals. Other morphological characteristics, such as the contrast of mitochondria, the presence of protrusions, the number of synapses made, and the postsynaptic targets were noted as categorical data when possible.

For model-based classification of soma sizes in geniculate laminae, the Gaussian normal mixture modeling feature of the R package *MClust* (Scrucca et al., 2016), which assumes that distinct subpopulations that make up a heterogeneous population are normally distributed, was used to determine the parameters of subpopulation clusters within the dataset of cell soma sizes across lamina. The selection of a model is determined by the highest Bayesian information criteria (BIC) between models of different cluster numbers. The *MClust* package then assigns a classification for every object into the determined clusters, along with each cluster's sample size, mean, and standard deviation, which are used to construct distribution curves of model subpopulations.

For three-dimensional object–based confocal colocalization analysis, we designed a new quantitative approach that accounts for object-size variabilities of boutons visualized by tracer-filling of axons versus the immunolabeling of multiple vesicle clusters within a single bouton: First, 3D renderings of terminal boutons were made using the “Surfaces” creation tool in the Imaris 9.5 software (Oxford Instruments). The surface detail was confined to 0.10 µm, and the intensity threshold was adjusted to define object boundaries. To minimize the inclusion of false objects created from background fluorescence, any rendering that was smaller than the volume of 15 voxels (0.0185 μm^3^) was excluded from analyses. For every object, volume and the centroid *X*/*Y*/*Z* coordinates were collected, and object radius was calculated from its volume. Object information was then funneled into the nearest neighbor search algorithm in the R package *FNN* (Arya et al., 1998) to obtain the ID of the nearest neighbor of each object and the Euclidean distance between the two objects. Interbouton distance was calculated by subtracting the radii of each object from the distance between them. Any two objects were deemed to be colocalized if their interbouton distance was <0. Percentages of VGluT2+ boutons that are not colocalized with a retinal bouton are calculated from five images from each lamina; each image has at least 200 boutons. To account for false-positive identification of objects, a colocalization index (CI) was calculated for each image by normalizing the percentage of VGluT2 objects containing CTB to the percentage of CTB objects containing VGluT2, as VGluT2 is assumed to be present in every retinal (i.e., CTB) terminal bouton. A CI value of 1.0 indicates that every VGluT2+ object also contained CTB, whereas a CI value of <1.0 indicates the presence of singly labeled VGluT2 objects.

Adobe Creative Cloud Photoshop was used for compiling figures, including pseudocoloring and annotation of EM images. For graphs and statistical analysis with nonparametric testing, including unpaired Mann–Whitney *U* (MW-*U*) tests, Kolmogorov–Smirnov (KS) and Kruskal–Wallis (KW) comparisons, and descriptive statistics, the GraphPad Prism software (GraphPad.com) was used. Table 2 lists all statistical parameters.

**Table 2.** Statistical parameters

Figure	Data structure	Type of test	*p* values	Power (95% CI)
1*h*	Nonparametric	MW-*U*	*p* < 0.0001***	Relay: 244.6–258.8, Int: 121.9–128.6
1*i*	Nonparametric	KW with multiple comparisons	*p* < 0.0001***	L1: 261.6–292.4 [Relay], 119.8–134.3 [Int]
				L2: 251.8–273.6 [Relay], 127.9–140.3 [Int]
				L3: 200.4–219.7 [Relay], 126.9–138.1 [Int]
				L4: 266.2–292.4 [Relay], 123.5–133.9 [Int]
				L5: 261.3–287.3 [Relay], 110.8–122.7 [Int]
				L6: 201.8–220.0 [Relay], 97.97–113.1 [Int]
1*j*	Nonparametric	KW with multiple comparisons	*p* < 0.0001***	L1: 261.6–292.4
				L2: 251.8–273.6
				L3: 200.4–219.7
				L4: 266.2–292.4
				L5: 261.3–287.3
				L6: 201.8–220.0
2*m*	Normal	One-way ANOVA, Tukey's multiple comparisons	*p* < 0.01* *p* < 0.001** *p* < 0.0001***	L1 vs L3: 27.17–111.1
				L1 vs L6: 36.12–120.0
				L2 vs L3: 28.92–100.5
				L2 vs L6: 37.88–109.4
				L3 vs L4: −96.62 to −25.08
				L3 vs L5: −79.26 to −7.724
				L4 vs L6: 34.03–105.6
				L5 vs L6: 16.68–88.22
3*c*	Nonparametric	MW-*U*	*p* < 0.0001***	L1: 0.3910–0.5454, 2.119–2.572
				L2: 0.4279–0.5882, 2.311–2.730
				L3: 0.5524–0.7879, 1.135–1.466
				L4: 0.4400–0.5677, 2.407–3.079
				L5: 0.4576–0.6044, 2.448–3.043
				L6: 0.4933–0.6565, 1.727–2.125
3*c* inset	Nonparametric	KW with multiple comparisons	*p* < 0.01*	L1–2: 0.5677–0.7248
				L3–6: 0.7670–0.9932
3*g*	Nonparametric	KS	*p* < 0.01* *p* < 0.001** *p* < 0.0001***	L1: 2.119–2.572
				L2: 2.311–2.730
				L3: 1.135–1.466
				L4: 2.407–3.079
				L5: 2.448–3.043
				L6: 1.727–2.125
4*d*	Nonparametric	KS	*p* = 0.0003**	L3: 1.135–1.466 [RG], 1.525–2.142 [LM]
5*e*	Nonparametric	MW-*U*	*p* < 0.0001***	L3: 1.235–1.591, 0.4360–0.5529
				L6: 1.492–1.893, 0.4545–0.7505
5*f*	Nonparametric	KS	*p* = 0.3871 *p* = 0.3155	L3: 1.235–1.591 [TG], 1.135–1.466 [RG]
				L6: 1.492–1.893 [TG], 1.727–2.125 [RG]
7*f*	Parametric	Welch's *t* test	*p* < 0.0001***	M/P: 1.002–1.113
				K: 0.5616–0.7411

*p < 0.01; **p < 0.001; ***p < 0.0001.

## Results

### Characterization of neurons in K versus M/P laminae

#### Relay cell differences across geniculate laminae

**Figure 1.**
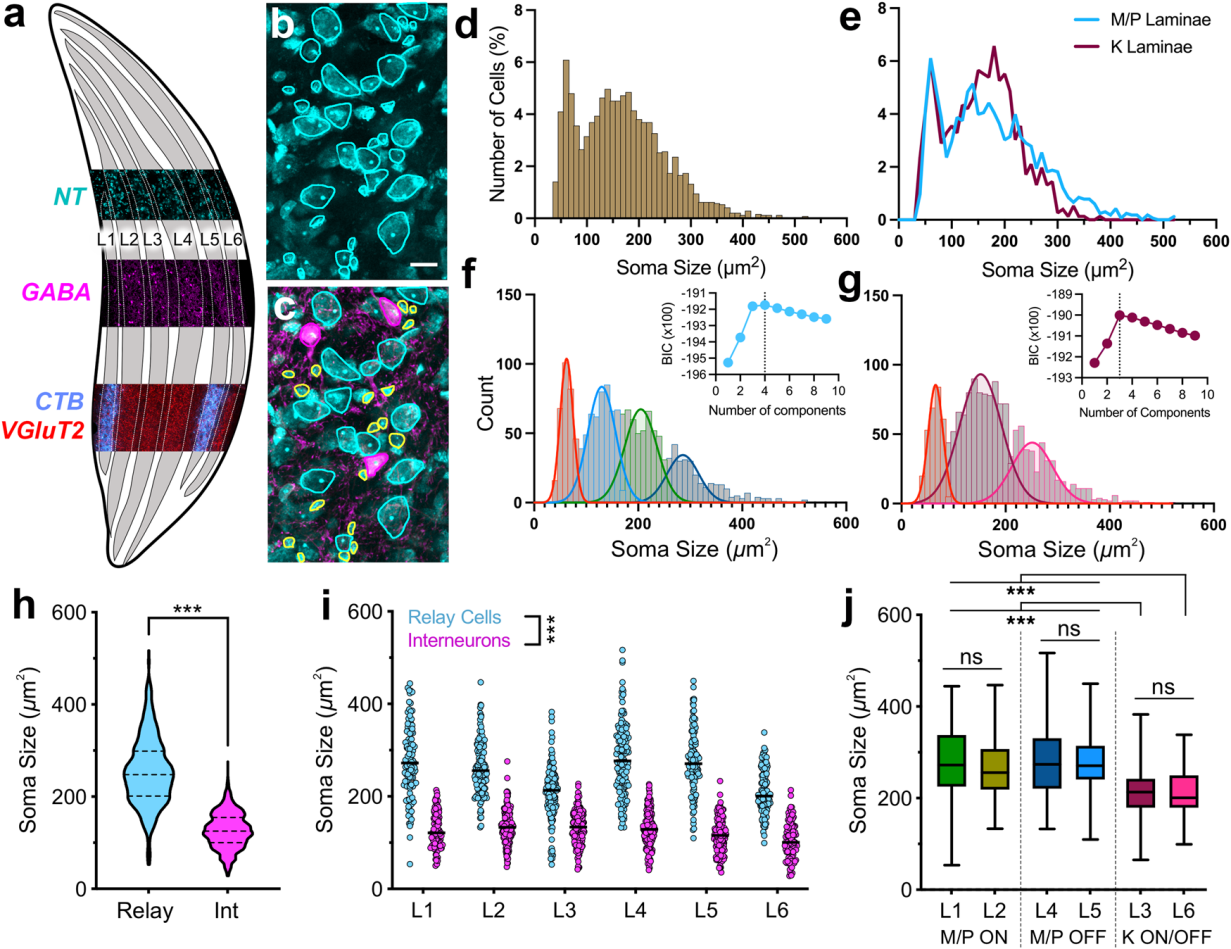
Cellular characteristics of M/P versus K dLGN laminae. ***a***, Schematic for delineation of geniculate laminae by anterograde tracers from eye injections (CTB, blue), immunostaining for VGluT2 (red), GABA (magenta), and NT (cyan). ***b***, ***c***, Sections dually stained for NT and GABA were used to quantify soma sizes of relay cells (NT + GABA−, cyan) and interneurons (NT + GABA+, magenta). Small glial cell bodies (yellow) were excluded from analyses. Scale bar, 20 μm. ***d***, ***e***, Frequency distribution histogram of soma sizes for all NT+ cells collected in L1–L6 that display two distinct peaks (***d***). When separated out by M/P (blue) versus K (maroon), the first peak, presumably interneurons, lines up, while larger-sized, relay cell populations reveal differences in their distributions (***e***). ***f***, Frequency distribution of soma sizes in M/P laminae with overlaid normal distributions of four subpopulations modeled via BIC. Inset shows the BIC for models with different numbers of components. The dashed vertical line marks the model with the highest BIC number. ***g***, Frequency distribution of soma sizes in K laminae with overlaid normal distributions of three subpopulations modeled via BIC. Inset shows the BIC for models with different numbers of components. The dashed vertical line marks the model with the highest BIC number. ***h***, Collapsed across lamina, all relay cells were significantly larger than all interneurons (*p* < 0.0001). ***i***, Distributions of soma sizes for relay cells and interneurons, sorted by lamina. In each lamina, relay cells are significantly larger than interneurons (*p* < 0.0001). ***j***, Relay cell soma size distributions, sorted by pathway. Within each lamina pair, there are no significant differences in the soma size. Relay cells in M/P L1/2 and M/P L4/5 are significantly larger from those in K L3/6. ***p < 0.0001.

In order to reveal if the cytoarchitectural differences across LGN laminae lend evidence for the cell-type specificity in M/P versus K pathways, we have quantified the sizes of cell somas that are outlined by neuronal markers. Retinogeniculate (RG) axons, labeled by an injection of CTB into the eye, as well as axon boutons stained for VGluT2, were used to delineate laminar borders (Fig. 1*a*). For measuring soma sizes, we weighed the advantages and limitations of a histochemical stain NeuroTrace (NT; Thermo Fisher Scientific) and the antibody based NeuN staining to visualize geniculate cells. While selective for neuronal nuclear protein, NeuN stain inherits limitations including dependence on antibody penetration and potential exclusion of some neuron types in the brain (Kumar and Buckmaster, 2007). On the other hand, NT reliably labels all cells with Nissl bodies, including glial cells (García-Cabezas et al., 2016). For our purposes, NT proved preferable because glial cell somas can be excluded based on size criteria. To that aim, we measured the sizes of geniculate GABA+ somata (*n* = 878; >100 cells per laminae; 125.2 ± 1.3 μm^2^; Fig. 1*h*) and determined that 99% of GABAergic cells were larger than 40 μm^2^, which is larger than glia measured in various species (Stolzenburg et al., 1989; Rajkowska et al., 1998; Verdonk et al., 2016; Liu et al., 2022). Any NT cells smaller than this cutoff value were classified as GABA+ glial cells and excluded from soma size analyses.

At exploratory size frequency distribution histograms (Fig. 1*d*,*e*), the NT+ neuron somata (*n* = 2,414) range between 40.2 and 523 μm^2^ and display two major peaks representing the size differences between interneurons and relay cells. When neurons in M/P and K laminae are plotted separately (Fig. 1*e*), the first, presumed interneuron, peaks align, whereas larger-sized, relay cell populations reveal nonoverlapping distributions with discrete peaks and thus potential differences.

To elucidate for the multiple cell subpopulations that contribute to distributions in M/P and K laminae, we used a statistical modeling approach, *Mclust*, which iteratively applies BIC to reveal the best fit number of normally distributed subpopulations that make up a multimodal distribution (Scrucca et al., 2016). MClust analysis revealed four distinct populations in M/P laminae and three distinct populations in the K laminae (Fig. 1*f,g*). When modeled distributions of MClust subpopulations were superimposed on the M/P and K laminae measured distributions, several findings were revealed: First, the smallest subpopulation in each laminae category completely accounts for the interneuron peaks, and these are not statistically different than each other. Thus, interneuron soma sizes in M/P and K laminae are similar. Second, M/P laminae are composed of three distinct cell types, potentially corresponding to M and P cell types in ON and OFF geniculate laminae of the tree shrew LGN with distinct soma morphology (Friedlander et al., 1981). Third, K laminae are also composed of two distinct cell types. Cell size distributions between K L3 and K L6 show similar trends and do not significantly differ, so this finding may support the existence of multiple K relay cell subtypes.

#### Comparison of relay cells and interneurons across geniculate laminae

In order to identify cell body differences across LGN laminae, we used histochemical and immunocytochemical markers to distinguish relay cells (NT+ and GABA−) from interneuronal (NT+ and GABA+) cell bodies (Fig. 1*b*,*c*). As relay cells are encountered more frequently than interneurons in the LGN, we employed a sampling strategy that aimed to collect at least 100 cells of each type from each lamina, thereby oversampling interneurons. Thus, the dataset is optimized to compare size differences across cell types and laminae, but it is not suitable for revealing the ratio of relay versus interneuron density in any given region. In a dataset of 1,592 cell somas (714 relay cells and 878 interneurons; >100 cells per lamina), the geniculate relay cells displayed significantly larger soma sizes than interneurons (251.7 ± 2.7 μm^2^ vs 125.2 ± 1.3 μm^2^; Fig. 1*f*; MW-*U*, *p* < 0.0001). The relay–interneuron soma size differences are also evident in each individual lamina (Fig. 1*i*; KW; *p* < 0.0001 for all laminar pairwise comparisons). Because the tree shrew LGN laminae are uniquely segregated in distinct pathways (L1 and L2, M/P ON; L4 and L5, M/P OFF; and L3 and L6, K ON/OFF), we asked whether pathway-specific differences in relay soma sizes exist within and across lamina pairs. We found no differences within M/P ipsi/contra pairs or within the contralateral K pair. However, relay cells in both ON and OFF M/P laminae are significantly larger from those in K laminae (Fig. 1*j*; KW; *p* < 0.0001; pairwise comparisons). Complimented by Mclust results above, this analysis confirms that M/P and K laminae contain distinct relay cell populations.

#### Calbindin and parvalbumin specificity across geniculate laminae

**Figure 2.**
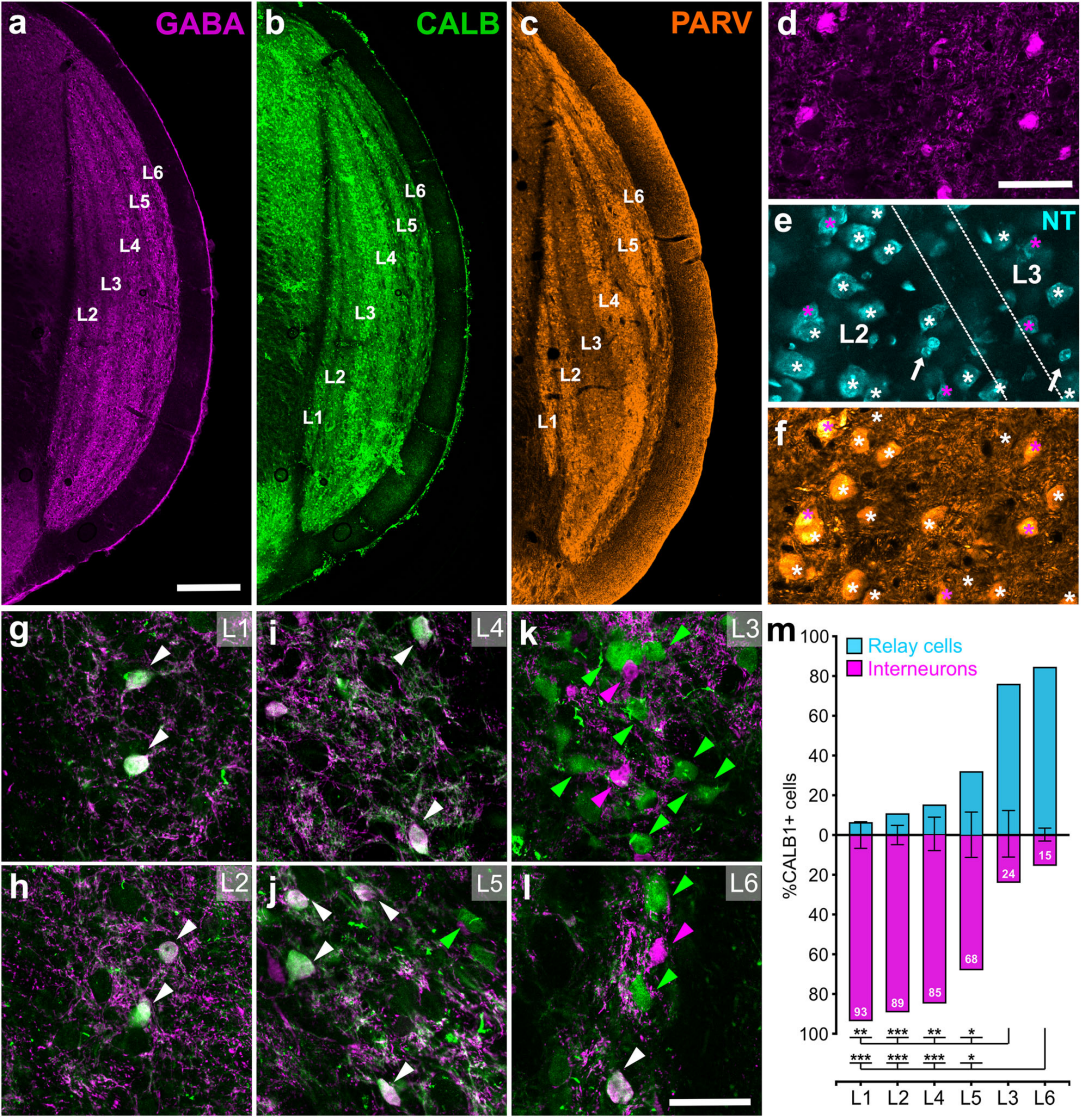
CALB, PARV, and GABA specificity of M/P versus K dLGN laminae. ***a–c***, GABA (magenta), CALB (green), and PARV (orange) staining across L1–L6. Scale bar, 500 μm. ***d–f***, Triple immunofluorescence staining reveals that PARV is present in all neuronal cell bodies, including non-GABAergic relay cells (white asterisks) and GABAergic interneurons (magenta asterisks), but not in small, glial cell types (white arrow) revealed by the Nissl stain (NT). Scale bar, 50 μm. ***g–l***. High (63×)-magnification views from each lamina, showing colocalization (white arrowheads) of GABA and CALB in M/P pairs (***g–j***) and K pair (***k***, ***l***). Scale bar, 50 μm. ***m***, The proportion of CALB+ cells that are relay cells versus interneurons in each lamina. Interneurons accounted for the majority of CALB+ cells in M/P lamina pairs, whereas relay cells accounted for the majority in the K lamina pair. *p < 0.01; **p < 0.001; ***p < 0.0001.

In order to further characterize the cell-type–specific markers in tree shrew dLGN, we quantified the distribution of the calcium-binding protein calbindin (CALB) and parvalbumin (PARV) across geniculate laminae in four tree shrews (Fig. 2*a*–*c*). Previously, CALB has been qualitatively described as a marker for K (or W)-type relay cells in tree shrews and primates (Jones and Hendry, 1989; Diamond et al., 1993; Johnson and Casagrande, 1995; Goodchild and Martin, 1998); however, the exclusivity of this staining neither to relay cells nor to K laminae was confirmed. Our quantitative laminar analysis revealed that CALB-stained cells are present in every lamina (Fig. 2*b*,*g*–*l*); thus, CALB is not exclusive to the K pathway. To elucidate differences in CALB+ relay cells and interneurons in these distinct pathways, the tissue was dually stained, CALB+ cells were sampled across all laminae in seven LGN tissues sections (*n* = 1,539), and the colocalization of GABA was assessed in those cells (Fig. 2*a*,*b*,*g*–*l*). K laminae displayed the majority of CALB+ cells (L3, 794; L6, 232) and ∼20% of those contained GABA, suggesting that CALB is utilized not only in relay cells but also in interneurons. In contrast, while CALB+ cells in M/P laminae were not as numerous (50–190 cells), 65–95% of CALB+ somata contained GABA (Fig. 2*g*–*i*,*m*), suggesting that CALB is indeed expressed in the M/P laminae and preferentially in interneurons (Fig. 2*m*). It should be noted that as these experiments did not include imaging of a third channel for all geniculate cells, they do not reveal whether all K relay cells, or all M/P interneurons, are CALB-positive. Regardless, they provide definitive confirmation that interneurons across all laminae contain CALB; however only relay neurons in K laminae predominantly express this calcium-binding protein.

PARV, a calcium-binding protein that is more prevalently specific for GABAergic neurons in the brain, has previously been described as a selective marker for M/P layers in both tree shrew and primates (Jones and Hendry, 1989; Diamond et al., 1993; Goodchild and Martin, 1998). In the current study, as expected, immunostaining with anti-PARV antibodies revealed denser staining in geniculate M/P laminae (Fig. 2*c*). However, this apparent selectivity at low magnifications was merely due to denser labeling of the neuropil in those laminae. Upon closer inspection we found labeling of cells in every geniculate lamina. Furthermore, PARV labeling was not confined solely to GABAergic cells (Fig. 2*d–f*); both GABA+/NT+ and GABA−/NT+ cells in all geniculate laminae were immunopositive for PARV. Thus, PARV is expressed in every neuron type in all geniculate laminae.

### Terminal bouton morphology of RG axons

#### Ultrastructural morphology of RG projections

**Figure 3.**
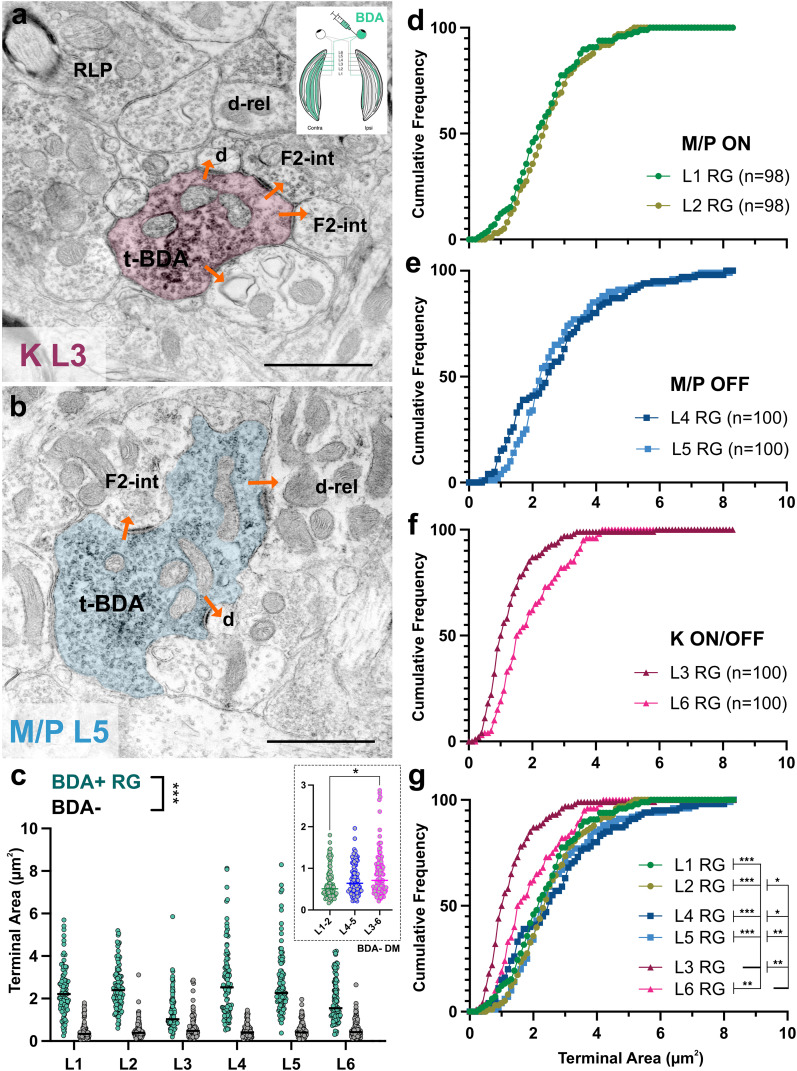
Retinal terminals in K L3 are distinct from all other geniculate laminae. ***a***, ***b***, Electron micrographs of RG terminals labeled anterogradely from the eye (t-BDA) in K L3 (***a***) and M/P L5 (***b***). RLP, unlabeled terminal with LM; d-rel, relay cell dendrite; F2-int, vesicle-containing interneuron dendrite; d, dendrite. Scale bar, 1 μm. ***c***, Quantitative comparison of terminal areas of labeled RG terminals (BDA+ RG) and unlabeled terminals (BDA−) in L1–L6. In every lamina, RG terminal cross-section areas are significantly larger than non-RG (*p* < 0.0001). The inset shows comparison of unlabeled terminals with DM across the three pairs of layers. BDA− DM terminals in the K pair show a greater range in sizes and are significantly larger than BDA− DM terminals in M/P L1–L2 (*p* < 0.01). ***d–f***, Cumulative frequency distributions of RG terminal areas in each pair of laminae. Within pairs, M/P L1–L2 (***d***) and M/P L4–L5 (***e***) have similar distributions, whereas K L3/L6 (***f***) significantly differ from one another (*p* < 0.001). ***g***, Comparison of distributions across lamina. K laminae significantly differ from M/P lamina, with L3 differing more so than L6. *p < 0.01; **p < 0.001; ***p < 0.0001.

In order to characterize the morphological differences of RGC terminals in M/P and K laminae, we examined LGNs after BDA injections into the eye using electron microscopy. Eye injections led to visualization of fibers in all ipsilateral or contralateral geniculate laminae, spanning the entirety of the topographic representation, suggesting that the spread of the tracer across the retina and the transport of tracer in RGC axons were near 100%. A total of 1,203 terminals that contained at least one synaptic zone at the cross section were examined; 596 of these were BDA-labeled RG terminals, and 607 were unlabeled terminals within the same regions. Retinal terminals labeled by the BDA injection exhibited a range of morphologies across all geniculate laminae. Most exhibited the typical morphology previously described (Guillery, 1969; Erişir et al., 1997; Bickford et al., 2010), exhibiting large cross-section areas and thick postsynaptic densities, making multiple synaptic contacts, containing many mitochondria and having protrusions (Fig. 3*a*,*b*).

#### Quantitative comparison of retinal terminals across geniculate laminae

In order to compare retinal terminals across geniculate laminae quantitatively, we measured the areas of RG terminals labeled after eye injections, as well as the areas of all unlabeled terminals in the same tissue. In all laminae, retinal terminals were the largest in size and were significantly larger than the unlabeled terminals within the same lamina (Fig. 3*c*; MW-*U*; *p* < 0.0001). However, interestingly, unlabeled terminals classified as having dark mitochondria (DM; nonretinal) in the contralateral K pair were the largest on average, ranging from 0.21 to 2.88 μm^2^ in size (Fig. 3*c*, inset) and significantly differed from one of the M/P ipsi/contra pairs (L3–L6, 0.88 ± 0.06 μm^2^ vs L1–L2, 0.65 ± 0.04 μm^2^; KW; *p* = 0.003; vs L4–L5, 0.75 ± 0.04 μm^2^, ns), consistent with the idea that K laminae receive an additional input that bear large terminal boutons that do not display light mitochondrion, the typical morphology of retinal axons.

Across geniculate laminae, the smallest-sized labeled retinal terminals resided in K-recipient L3 (1.30 ± 0.08 μm^2^), and the largest-sized labeled retinal terminals resided in M/P-recipient L5 (2.75 ± 0.15 μm^2^). The pair of M/P-recipient ON layers did not significantly differ from one another (Fig. 3*d*; L1 RG terminals, 2.35 ± 0.11 μm^2^; L2 RG terminals, 2.52 ± 0.11 μm^2^). The other pair of M/P-recipient OFF layers also did not significantly differ from one another (Fig. 3*e*; L4 RG terminals, 2.74 ± 0.17 μm^2^; L5 RG terminals, 2.75 ± 0.15 μm^2^). However, the pair of K-recipient ON/OFF layers were the only pair that significantly differed from one another (Fig. 3*f*; L3 RG terminals, 1.30 ± 0.08 μm^2^; L6 RG terminals, 1.93 ± 0.10 μm^2^; *p* < 0.0001), suggesting that the two K laminae may be involved in distinct functions. Additionally, retinal terminals in K-recipient L3 significantly differed from every other lamina (Fig. 3*g*; *p* < 0.001–0.0001). Similarly, the other K-recipient lamina L6 also significantly differed from every other layer, except M/P L1, but to a lesser extent (Fig. 3*g*; *p* < 0.01–0.001). Together, these results reveal that retinal terminals in M/P lamina pairs are more similar to each other than they are to the K pair and that retinal terminals in K-input laminae are distinct from all other geniculate laminae.

### Ultrastructural morphology of geniculate terminals

**Figure 4.**
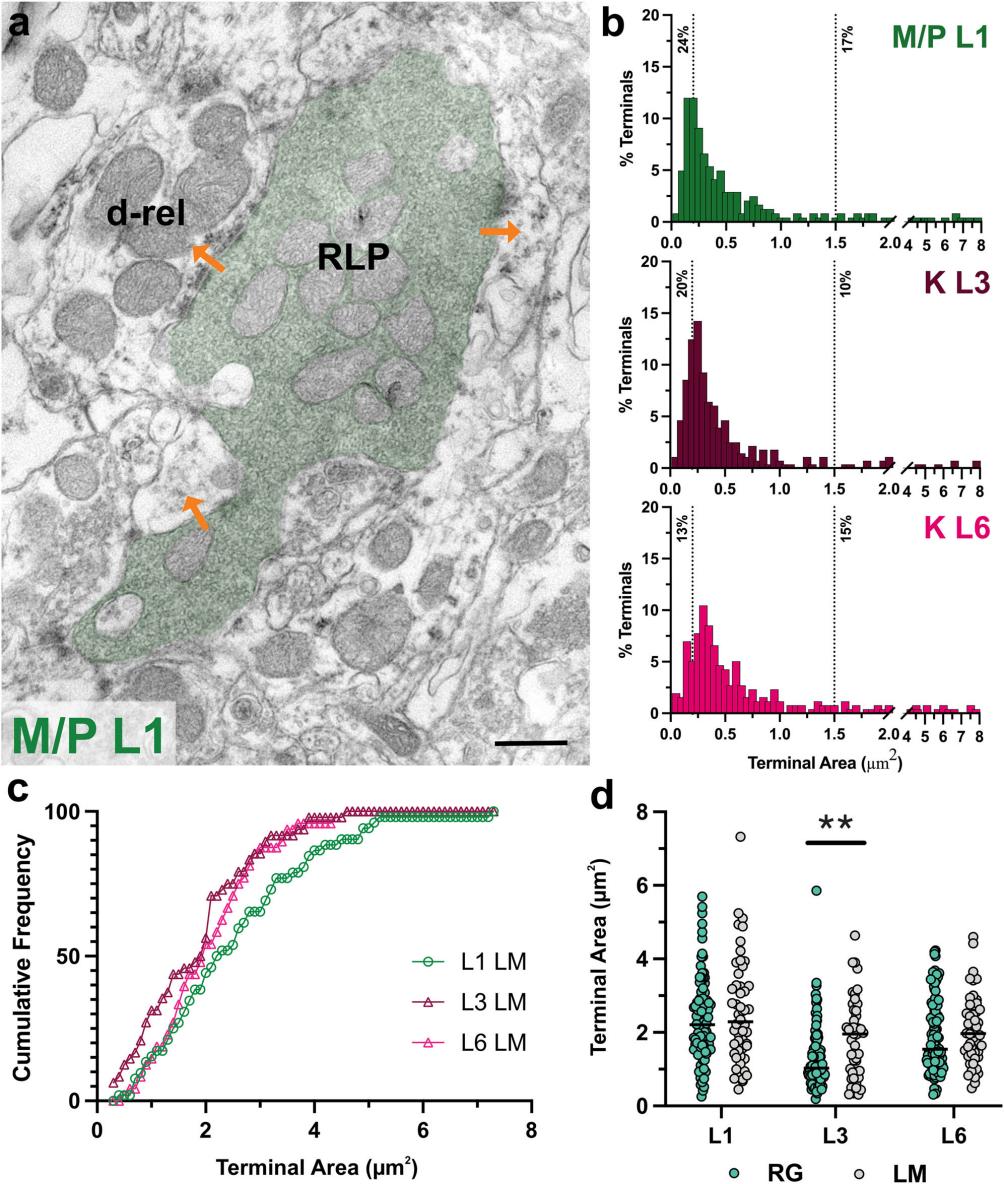
Differences in input contribution in M/P versus K laminae. ***a***, Electron micrograph of a large terminal, identified by its LM (RLP), making multiple synapses, including onto a relay cell dendrite (d-rel). Scale bar, 1 μm. ***b***, Frequency distributions of terminal cross-section areas in M/P L1 (top, green), K L3 (middle, maroon), and K L6 (bottom, pink). Dashed lines and percentages indicate the proportion of terminals smaller than 0.20 μm^2^ and the proportion of terminals larger than 1.50 μm^2^. M/P L1 contains a greater percentage of large-sized terminals, typically of retinal origin, while K L6 contains fewer of the smallest-sized terminals, typically originating from the cortex. ***c***, Cumulative frequency distributions of terminal areas of presumed retinal terminals, identified by their LM, in M/P L1, K L3, and K L6. LM terminals were the smallest in L3. ***d***, Comparisons of terminal area distributions between tracer-identified retinal terminals (RG) and presumed retinal terminals identified by LM. LM terminals only differed significantly from RG terminals in K-recipient L3 (*p* < 0.001). **p < 0.001.

To capture the laminar differences in geniculate synaptic circuitry contributed by all projections, we used unlabeled tissue sections collected from one animal and examined the synaptic connections of a total of 781 terminals. We measured 242 synaptic terminals in M/P-recipient L1 (Fig. 4*a*; 0.84 ± 0.07 μm^2^), 281 synaptic terminals in K-recipient L3 (0.61 ± 0.04 μm^2^), and 258 synaptic terminals in K-recipient L6 (0.77 ± 0.05 μm^2^). Frequency distributions of cross-section areas in these laminae suggest potential differences in contribution of input subpopulations with unique sizes: M/P-recipient L1 contains a greater percentage of large-sized terminals, especially compared with K-recipient L3, while K-recipient L6 contains fewer of the smallest-sized terminals, typically originating from cortex (Fig. 4*b*).

On nonimmunolabeled EM preparations, putative retinal and nonretinal terminals are traditionally identified based on whether they contain light mitochondria or DM (Erişir et al., 1998; Bickford et al., 2015). We applied the same approach to examine laminar differences among terminals that meet this morphological criterion. While unlabeled terminals with light mitochondria (LM) displayed the smallest mean in K-recipient L3, no statistical differences in their size distribution were evident when compared with L1 (Fig. 4*c*; 1.83 ± 0.15 μm^2^ vs 2.55 ± 0.20 μm^2^; KS, *p* = 0.10) or L6 (2.08 ± 0.14 μm^2^; *p* = 0.37). However, terminals identified as retinal based on their pale mitochondrial appearance differed significantly from tracer-identified RG terminals in K-recipient L3 (Fig. 4*d*; L3 LM terminals 1.83 ± 0.15 μm^2^ vs L3 RG terminals 1.30 ± 0.08 μm^2^; *p* < 0.001). This suggests that either retinal terminals do not account for all terminals with LM or that mitochondrial appearance is not a reliable criterion for retinal terminal identification, at least in K L3.

### Terminal bouton morphology of TG axons

#### Ultrastructure of TG projections

**Figure 5.**
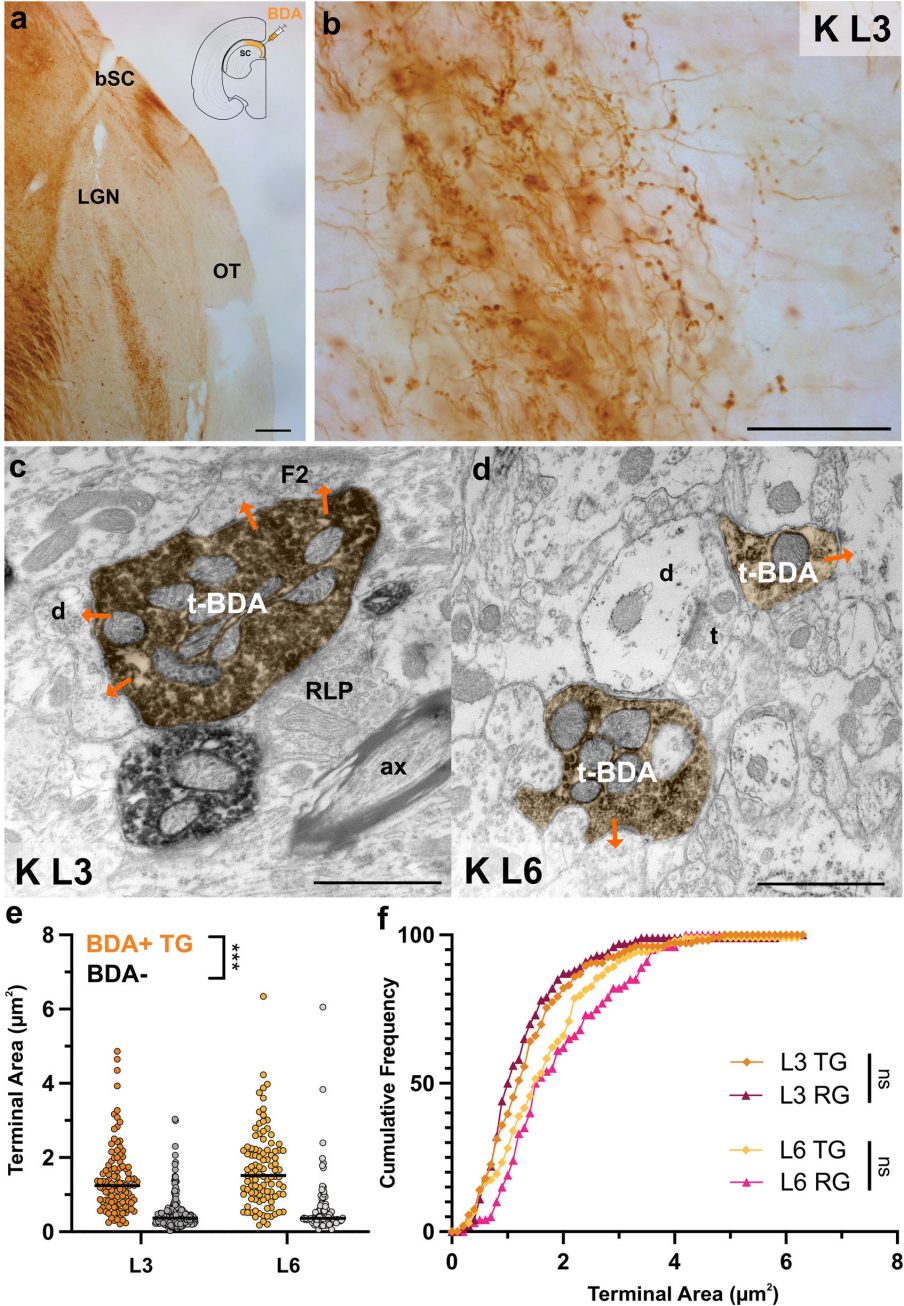
TG terminals in K L3 and L6 have retinal-like morphology. ***a***, Anterogradely labeled TG axons traveling in the brachium of SC (bSC) and optic tract (OT) to terminate in the LGN at 5× magnification. Scale bar, 100 μm. ***b***, TG axon bouton terminals in K L3 shown at 40× magnification. Scale bar, 50 μm. ***c***, ***d***, Electron micrographs of large, labeled TG terminals (t-BDA) in K L3 (***c***) and K L6 (***d***). RLP, unlabeled retinal terminal with LM; ax, myelinated axon; F2-int, vesicle-containing interneuron dendrite; d, dendrite; t, unlabeled terminal. Scale bar, 1 μm. ***e***, Quantitative comparison of terminal areas of labeled TG terminals (BDA+ TG) and unlabeled terminals (BDA−) in L3 and L6. In both K lamina, TG terminal cross-section areas are significantly larger than BDA− terminals (*p* < 0.0001). L3 TG terminal cross-section areas do not differ significantly from L6 TG terminals. ***f***, Cumulative frequency distributions of labeled TG and RG terminal areas in K L3 and K L6. In each lamina, TG terminals do not differ significantly from RG terminals. ***p < 0.0001.

BDA tracer injections into the SC or brachium of SC yielded robust labeling of TG axons with beaded appearance in both K L3 and L6 (Fig. 5*a*,*b*). Unlike previous studies (Fitzpatrick et al., 1980; Harting et al., 1991), our SC injections did not reveal many fibers in the interlaminar regions other than a few isolated axons on route from the optic tract to L3; the interlaminar tectal axons are not regarded further. Also to note, we observed no labeled axons in M/P L1–L2 or L4–L5. This finding argues against the possibility that our SC injections retrogradely filled M/P retinal ganglion cell axon collaterals, as occurs with Y-retinal axons in the cat (Tamamaki et al., 1995; Datskovskaia et al., 2001). Consequently, we ruled out the possibility that retrogradely filled RGC axon collaterals intermingle with TG axons in the K laminae of the tree shrew LGN.

We examined morphological properties of a total of 526 synaptic terminals (209 labeled TG terminals and 317 unlabeled terminals in the same sections as the labeled TG terminals). Most TG terminals in both K-recipient L3 and L6 were medium to large in size and significantly differed from the unlabeled (BDA−) terminals collected from the same lamina (L3 TG terminals 1.41 ± 0.09 μm^2^ vs L3 unlabeled terminals 0.49 ± 0.03 μm^2^; *p* < 0.0001; L6 TG terminals 1.69 ± 0.10 μm^2^ vs L6 unlabeled terminals 0.60 ± 0.07 μm^2^; *p* < 0.0001). The morphological characteristics of TG terminals resembled those originating from the retina (Fig. 5*c*,*d*), as they contained many mitochondria, made multiple synaptic contacts, and had protrusions, suggesting that TG axons may act as functional drivers in K laminae of LGN. Whether or not TG terminals contain light mitochondria or DM could not be assessed unambiguously because DAB chromogen in terminals could confound the mitochondria contrast.

#### Quantitative comparison of RG and TG projections

In both K-recipient laminae, TG terminals were significantly larger than the unlabeled terminals within the same lamina (Fig. 5*e*; MW-*U*; *p* < 0.0001). TG terminals in K-recipient laminae did not differ significantly from one another (Fig. 5*e*), unlike their RG counterparts (Fig. 3*f*,*g*). In L3, anterogradely labeled TG terminals were found to not differ significantly from RG terminals collected from the same laminae (Fig. 5*f*; L3 TG terminals 1.41 ± 0.09 μm^2^ vs L3 RG terminals 1.30 ± 0.08 μm^2^). Our analysis also revealed no significant difference in the size distributions of TG terminals from RG terminals collected in L6 (Fig. 5*f*; L6 TG terminals 1.69 ± 0.10 μm^2^ vs L6 RG terminals 1.93 ± 0.10 μm^2^).

**Figure 6.**
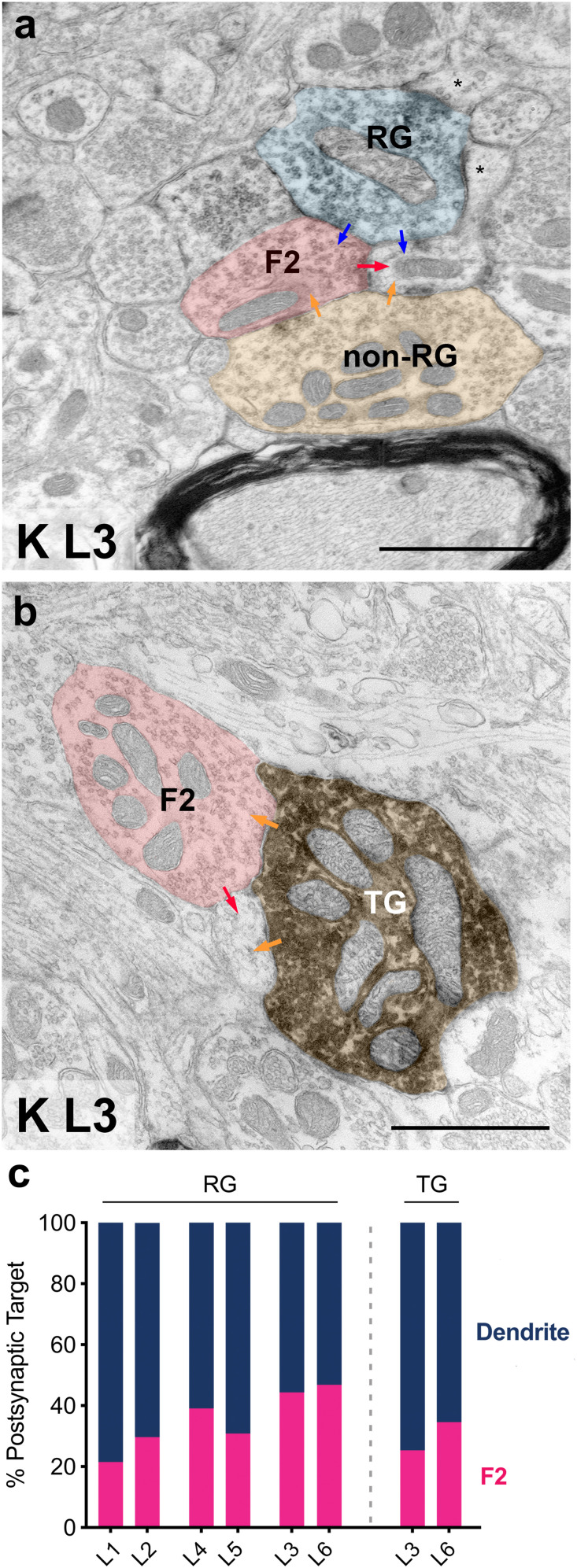
Tectal inputs mimic driver-like properties of retinal terminals. ***a***, Electron micrograph of a labeled RG terminal and an unlabeled, DM terminal (non-RG), presumed TG, converging on the same dendrite and forming triadic arrangements with a vesicle-containing interneuron dendrite (F2). Scale bar: 1 μm. ***b***, Electron micrograph of a labeled TG terminal forming a triadic arrangement with a vesicle-containing interneuron dendrite (F2). Scale bar, 1 μm. ***c***, Proportion of postsynaptic targets for RG and TG terminals. TG terminals contacted F2s, but to a lesser degree compared with RG terminals in the same lamina pair (L3, L6).

In order to further ascertain if tectal inputs mimic driver-like properties of retinal inputs, we characterized the postsynaptic target of every recorded synapse across both RG and TG boutons. Postsynaptic targets were either classified as a dendrite, which included thin appendages and protrusions, or a vesicle-filled profile (presumed F2). This analysis revealed that, similar to the RG terminals, TG terminals engaged in triadic arrangements (Fig. 6*a*,*b*), although less frequently compared with RG boutons in the same K laminae (Fig. 6*c*). However, considering both RG and TG axons innervate L3 and L6, this suggests that K laminae may contribute to local, inhibitory circuitry more than their M/P counterparts.

**Figure 7.**
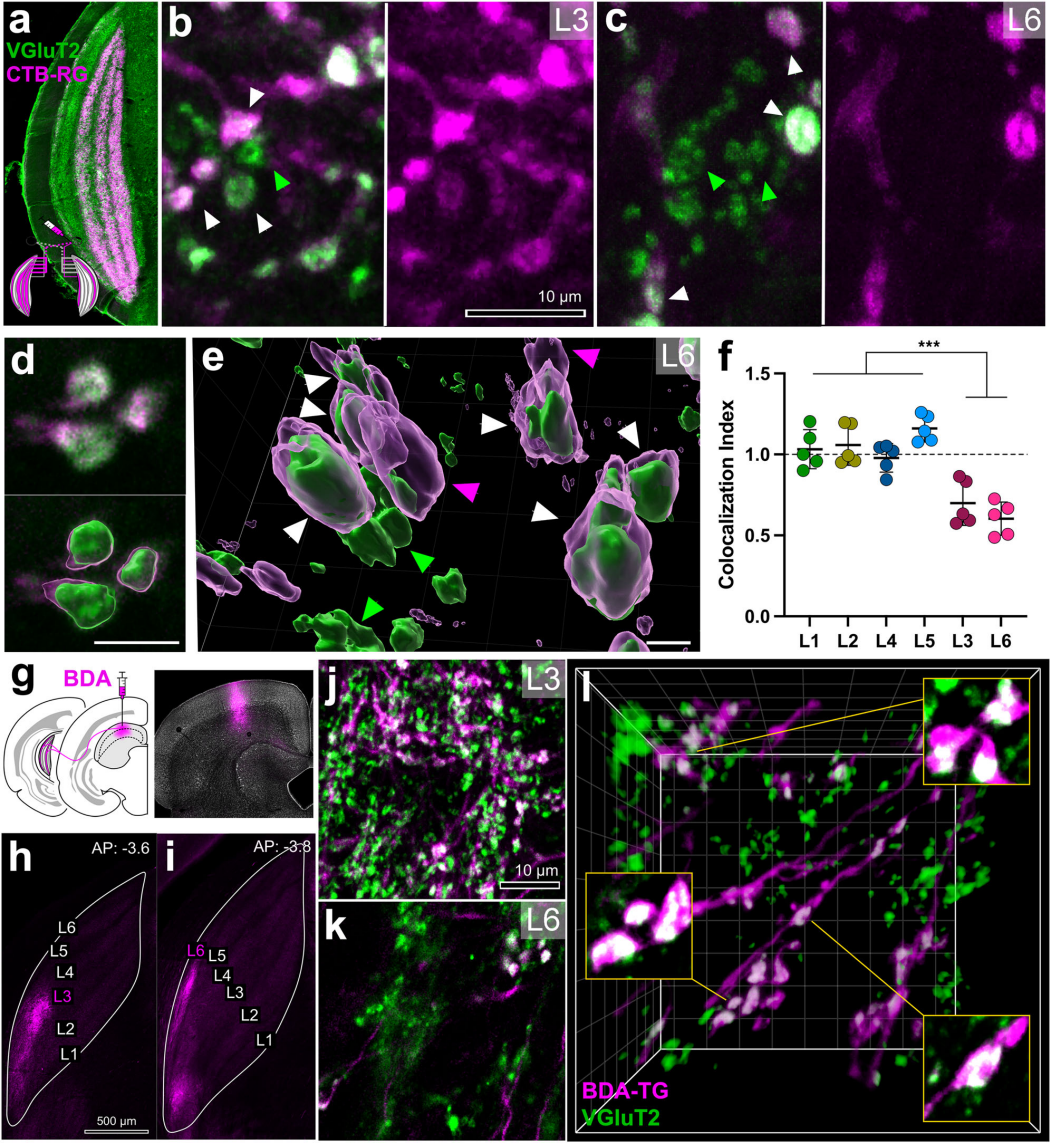
The K pathway receives an extra VGluT2-positive input from the SC. ***a***, LGN immunostained for VGluT2 (green) and contralateral RG projections labeled by CTB (CTB-RG, magenta). ***b***, ***c***, Dually labeled LGN sections in L3 (***b***) and L6 (***c***) showing colocalization between VGluT2+ boutons and anterogradely filled retinal terminals (white arrowheads) and singly labeled VGluT2 boutons nonretinal in origin (green arrowheads). Scale bar, 10 μm. ***d***, Example of dynamic volume rendering of three colocalized boutons in the Imaris software. Scale bar, 2 μm. ***e***, Example field of 3D-rendered boutons in L6 revealing VGluT2 boutons surrounded by CTB (white arrowheads), retinal boutons not containing VGluT2 (magenta arrowheads), and singly labeled VGluT2 boutons (green arrowheads). Scale bar, 2 μm. ***f***, CI of CTB+ and VGluT2+ objects in L1–L6. M/P laminae have CI values closer to 1.0 than K laminae, indicating that K laminae have a greater prevalence of singly labeled VGluT2+ objects that are not retinal in origin. ***g***, Schematic of BDA injection into SC (left) and labeled needle track at injection site (right). ***h***, ***i***, Labeled projections in K L3 (***h***) and K L6 (***i***) in dLGN of the thalamus. ***j–l***, The LGN tissue stained for VGluT2 (green) and anterogradely filled fibers and boutons (BDA-TG, magenta). There was overlap between VGluT2+ boutons and TG terminals in both K L3 (***j***) and K L6 (***k***), and BDA-TG boutons were filled substantially with VGluT2 (white, ***l***). Scale bar, 10 μm. ***p < 0.0001.

To determine whether TG terminals utilize the same glutamate transporter as retinal driver terminals, we injected CTB in one eye to label RG axons in the LGN and then costained the sections with VGluT2, which strongly labeled and delineated all six laminae (Fig. 7*a*). The *Z*-scans of dually labeled laminae revealed CTB-filled axons with their swellings, as well as VGluT2+ boutons (Fig. 7*b*,*c*; note that VGlutT2 rarely fills axonal fibers, which are devoid of vesicles). Using the Imaris software to analyze the *Z*-scans, we identified all potential VGluT2+ regions, assessed the colocalization of CTB+ pixels within these regions, and observed areas with only VGluT2 labeling (Fig. 7*b*,*c*). Particularly in L6, clusters of VGluT2+ regions were apparent.

Because confocal images do not provide detailed information on discrete bouton morphologies—especially with VGluT2 immunolabeling—and to assess the prevalence of VGluT2 labeling outside of retinal axons, we devised an object-based quantification approach using dynamic volume rendering of fluorescently labeled boutons (Fig. 7*d*,*e*). Assuming that the absence of VGluT2 in CTB+ terminals represents background noise or false-negative colocalization, we calculated a CI for each image by normalizing the percentage of CTB localization within VGluT2-labeled regions to the percentage of VGluT2 localization within CTB-labeled regions. An index value of 1.0 indicates complete colocalization, whereas values below 1.0 suggest the presence of VGluT2 boutons that are not colabeled with CTB. The M/P laminae exhibited indices close to 1 (1.06 ± 0.03), while the K laminae showed lower values (0.65 ± 0.04; Fig. 7*f*), and these two groups were significantly different from one another (Welch's *t* test; *p* < 0.0001). While there were more singly labeled VGluT2 boutons in L6 (L3 0.70 ± 0.06 vs L6 0.60 ± 0.05), indicating differences between two K laminae, a statistical difference was not evident (Welch's, *p* = 0.25). These results indicate that L3 and L6 display a higher prevalence of VGluT2 boutons that are not of retinal origin.

To test whether TG axons may account for the nonretinal VGluT2+ boutons in L3 and L6, we injected the anterograde tracer BDA into the superficial SC and visualized the TG axons using a streptavidin fluorescence procedure (Fig. 7*e*–*h*) on sections dually labeled for VGluT2, VGluT1, and GABA. We found no colocalization of VGluT1 or GABA with anterogradely transported BDA in TG boutons (data not shown) in either L3 or L6. On the other hand, qualitative evidence was present for VGluT2-ir colocalization with BDA in both K L3 and L6 (Fig. 7*i*–*o*). Quantitative assessment for the rate of VGluT2/SC axon colocalization was not feasible, because, unlike for the assessment of VGluT2/retinal axon described above (Fig. 7*f*), a background normalization condition, where 100% colocalization could be expected, was not present in SC injection experiments given that axons only project to K laminae. We should also note that in similar experiments where we used AAV-CAG-tdTomato as the anterograde tracer of TG axons, we failed to demonstrate any colocalization, underscoring that viral transfection may sometimes occlude any subsequent immunolabeling (Watakabe et al., 2015).

**Figure 8.**
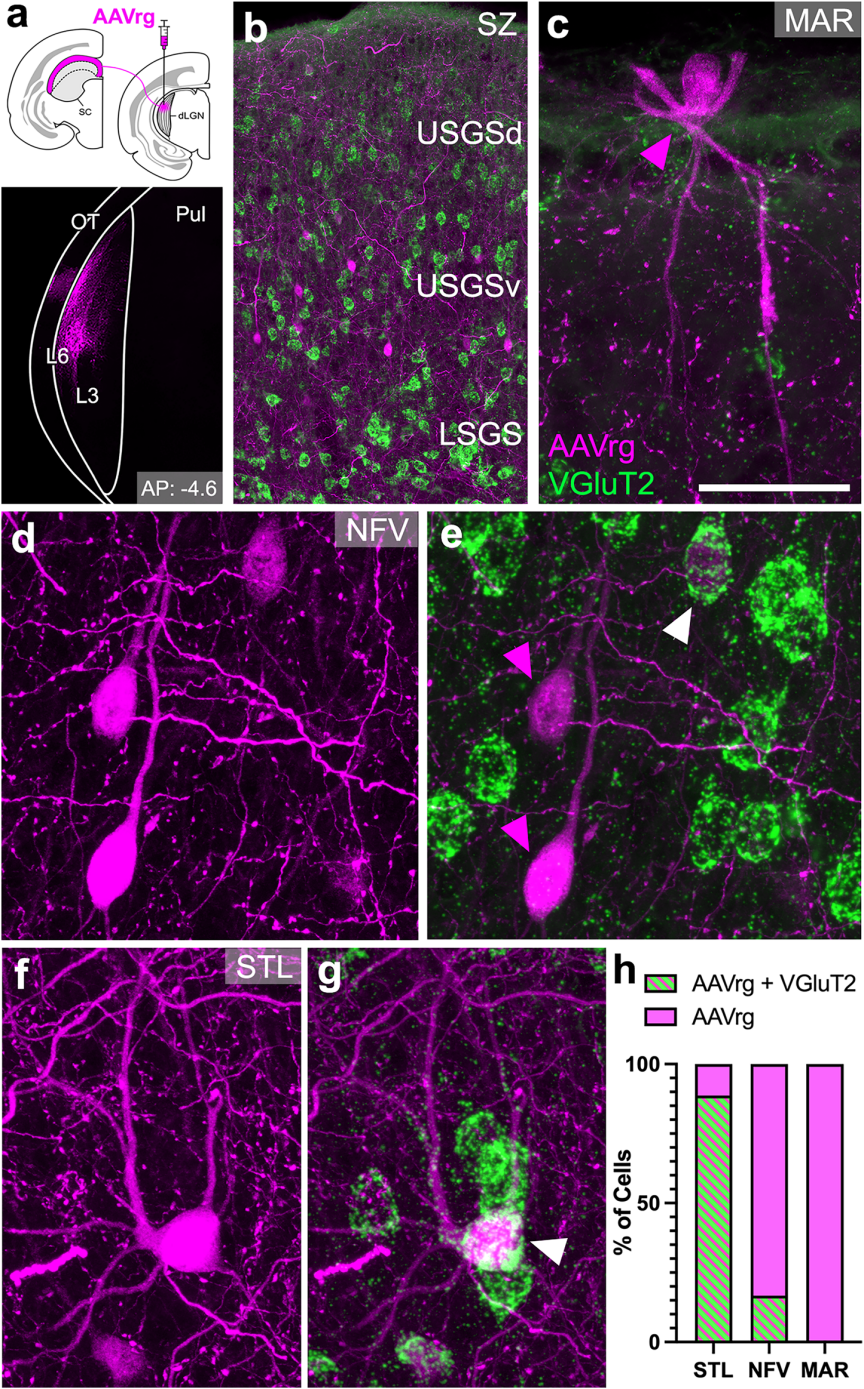
Three tectal cell types project to dLGN, but only one may account for the VGluT2-positive input. ***a***, Schematic of AAVrg injection into dLGN (top) and labeled injection site that is confined to dLGN and spans across L3 and L6 (bottom). ***b***, Retrogradely filled cells (magenta) shown in the upper superficial gray (SGS) of the SC, with most cell bodies residing in the ventral upper SGS (USGSv) and none in the lower superficial gray (LSGS), where pulvinar-projecting cells reside. SZ, zonal layer; USGSd, dorsal upper superficial gray. ***c***, Retrogradely filled marginal cell (MAR, magenta arrowhead) with a small soma at the dorsal surface of the SC that does not contain VGluT2 mRNA transcript (green). Scale bar, 1 μm. ***d***, ***e***, A field of retrogradely cells with fusiform somata and bipolar dendrites, suggestive of NFV cell morphology (***d***). Two of these NFV-like cells do not contain VGluT2 transcript (magenta arrowheads), while another is positive for VGluT2 FISH signal (white arrowhead, ***e***). ***f***, ***g***, Retrogradely filled stellate cell with a round soma and radially emerging dendrites (STL, ***f***) that contains VGluT2 FISH signal (white arrowhead, ***g***). ***h***, Quantitative comparison of prevalence of VGluT2 transcript across identified cell types. VGluT2 transcript (green striped bar) was present most prominently in stellate cells (STL), whereas the majority of NFV-like and marginal (MAR) cells did not contain VGluT2 mRNA (magenta bars).

To confirm the presence of VGluT2 transcript in SC neurons that project to the LGN, we stereotaxically injected the retrograde tracer AAV-CAG-GFP into the LGN (Fig. 8*a*) and then detected VGluT2 transcript using RNA-FISH. Injections confined to the geniculate laminae produced retrograde labeling in cells located in the upper superficial gray (SGS) and the zonal layer (Fig. 8*b*), while no labeled cells were observed in the lower SGS, where pulvinar-projecting wide–field vertical cells reside. The geniculate-projecting cells exhibited three distinct morphologies. Stellate cells—with round somata and radially emerging primary dendrites (Fig. 8*f,g*)—were the most common, accounting for ∼40% of labeled cells encountered across three SC sections (*n* = 131). Cells with fusiform somata oriented perpendicular to the surface, which gave off bipolar dendrites and are suggestive of narrow-field vertical (NFV) cell morphology (Fig. 8*d*,*e*), comprised 27.5% of retrogradely labeled cells. Finally, marginal cells with small somata found immediately ventral to the SC pial surface and gave off fine, ramifying dendrites (Fig. 8*c*), made up 22.9% of the geniculate-projecting neurons. These findings are consistent with previous descriptions of geniculate-projecting tectal cells in the tree shrew (Albano et al., 1979; Graham and Casagrande, 1980; Diamond et al., 1991; Harting et al., 1991).

RNA-FISH labeling revealed that VGluT2 transcript was most prominent in stellate cells, with 88.7% of these cells expressing VGluT2 (Fig. 8*h*). In contrast, only 16.7% of NFV-like cells and none of the marginal cells expressed VGluT2 mRNA (*n* = 36 and 30, respectively). Thus, TG projections in the tree shrew are morphologically and molecularly heterogeneous: three distinct cell types contribute to the TG terminals in the K geniculate laminae, but only one appears to provide most of the VGluT2-positive input. However, because our retrograde tracer injections spanned all geniculate laminae, our experiments cannot determine whether any of these three tectal cell types preferentially project to L3 or L6.

## Discussion

Our analysis of anatomical properties of M/P and K laminae of the tree shrew LGN revealed: (1) Relay cells in K-recipient laminae are morphologically and molecularly distinct from those in the Magno/Parvo-recipient laminae: they have smaller somata and contain CALB, suggesting that morphological distinctions in RGCs are preserved through parallel pathway-specific channels in LGN; (2) calcium-binding proteins display interesting patterns of specificity; CALB is expressed in GABAergic cells across all laminae, in addition to being a marker for K laminae relay cells. In contrast, PARV is not specific to any neuron type or laminae. This suggests that tree shrew relay cells differ neurochemically in their CALB, but not PARV, expression in the LGN; (3) RGC axon terminals projecting to the Magno/Parvo versus K-recipient laminae are morphologically distinct: K retinal driver terminals, especially in L3, are significantly smaller, which is consistent with innervation by distinct phenotypes of RGC axons in these pathways; (4) TG axons, which originate from three distinct cell types in the SC, closely resemble retinal terminals and many are VGluT2-positive, suggesting that TG axons may act as functional drivers in the K laminae of the tree shrew LGN.

### Morphological distinction of the K pathway

Parallel pathways of the vision originate from structurally and functionally distinct ganglion cells of the retina (Rodieck and Watanabe, 1993; Dacey, 2000; Dacey and Packer, 2003; Callaway, 2005). Similar to their putative retinal ganglion cell counterparts, relay cells in X/Y/W or M/P/K geniculate laminae display differences in soma sizes (LeVay and Ferster, 1977; Friedlander et al., 1981), and these differences were also evident in soma sizes of the tree shrew K lamina relay cells in our study. In addition, our study has brought clarification to whether or not M/P/K RGC axon terminals can be morphologically distinct. While the prototypical geniculate retinal terminal, as described by Guillery (1969), displays strikingly large cross sections in all mammalian species studied to date and in fact constitutes the largest-sized terminal boutons (Erişir et al., 1997, 1998; Van Horn et al., 2000), the studies of their size distribution have revealed that a wide range existed (Erişir et al., 1997, 1998). In fact, a more recent study revealed that multiple subpopulations make up the range of retinal bouton volume distributions (Maher et al., 2023), suggesting that pathway-specific RGC axons may provide morphologically distinct retinal boutons in the geniculate circuitry. With the unique advantage of segregated pathways of the tree shrew LGN, the current study now provides evidence that retinal terminals synapsing in the K laminae constitute the smaller-sized subpopulation.

### Neurochemical distinction of the K pathway

A key argument for classifying K cells as a separate group arose from evidence demonstrating that the K pathway is neurochemically distinct from the M and P pathways, particularly in the expression patterns of calcium-binding proteins. In nonhuman primates and tree shrew, researchers observed that many cells in K-recipient LGN, but only a few in M/P-recipient laminae, displayed immunoreactivity for CaMKII (Benson et al., 1991; Hendry and Jones, 1991; Hendry and Yoshioka, 1994) and CALB (Jones and Hendry, 1989; Diamond et al., 1993; Hendry and Yoshioka, 1994; Johnson and Casagrande, 1995; Goodchild and Martin, 1998), suggesting that CALB expression can be a selective marker of K lamina cells (for review: Hendry and Reid, 2000). An omission in these studies has been the characterization of CALB cells that sparsely yet persistently appear in M/P laminae. We have advanced this understanding in the tree shrew demonstrating that, while K relay cells are neurochemically distinct from M/P relay cells through their selective expression of CALB, GABAergic cells across all geniculate laminae, including the K laminae, also express CALB. Furthermore, our results revealed that, unlike in the 1993 study in the tree shrew (Diamond et al., 1993) or in primates (Jones and Hendry, 1989; Diamond et al., 1993; Goodchild and Martin, 1998), immunostaining with PARV antibodies visualized cells indiscriminately across all laminae, suggesting that PARV staining was not a reliable marker for M/P relay cells in the tree shrew.

Why may it be of importance for relay cells in the K pathway to selectively express CALB? Given the role of calcium-binding proteins as a second messenger activated by increases in intracellular calcium, their role in synaptic transmission and plasticity has been well established (Blatow et al., 2003; Catterall and Few, 2008; Westerink et al., 2012). The LGN has long been regarded as a rigid, unmodifiable relay center with the assumption that plasticity typically occurs in the cortex. However, studies have shown plasticity at the RG synapse in the mature LGN, partially due to postsynaptic processes of differential regulation of AMPA and NMDA receptors (Chen et al., 2002) and Ca^2+^-dependent postsynaptic processes. Similarly, metabotropic glutamate receptors, in particular, mGluR1 and mGluR5 which are coupled to phosphatidylinositide hydrolysis and intracellular calcium mobilization, are expressed differentially in triadic relay dendrites and presynaptic appendages of interneurons (Godwin et al., 1996). While the presence of CALB and PARV in geniculate interneurons is consistent with mGluR5-mediated intracellular calcium-activated processes, the specificity of CALB in K-type relay cells indicate a selective capacity of plasticity in geniculate K-type relay cells.

### Are there multiple K pathways?

Functional evidence supports an heterogeneity of K-type relay cells (Norton and Casagrande, 1982; Irvin et al., 1986; Norton et al., 1988; Holdefer and Norton, 1995; White et al., 1998, 2001; Eiber et al., 2018). K cells exhibit diverse response properties: some show unique functional signatures, such as blue- on or suppressed-by-contrast responses (Tailby et al., 2007; Roy et al., 2009; Jayakumar et al., 2013), some display direction selectivity or visual properties absent in M/P cells (Solomon et al., 2002), and other K cells respond minimally or not at all to traditional visual stimuli (White et al., 2001; Xu et al., 2001). Additionally, two distinct groups of W cells in the cat retina were distinguished based on the soma size and firing patterns (Stanford et al., 1981; Stanford, 1987), and retinal terminals in W-cell recipient zones of the cat LGN display morphological heterogeneity (Chen et al., 1996). In the macaque, there's also evidence for morphological distinctions of corticothalamic feedback neurons within different parallel processing streams (Hasse et al., 2019). While our results in the tree shrew do not reveal heterogeneity in neurochemical markers and relay cell soma sizes within K laminae, morphological properties of synaptic inputs in two K laminae suggest key differences. First, our terminal size analyses in K-recipient LGN laminae revealed two morphologically distinct populations, with K L3 having smaller retinal terminals than K L6, suggesting at least two distinct RGC types innervate these two laminae selectively. Second, the K laminae pair displayed evidence of distinct circuitries: analysis of postsynaptic targets of RG inputs showed that K L6 had a higher incidence of engagement in triadic arrangements compared with L3. Additionally, K L6, but not K L3, was impoverished of small-sized glutamatergic terminals (presumed corticothalamic) that constitute up to one-third of synaptic inputs to geniculate laminae, suggesting major differences in feedback excitation that impinges on cells in two K laminae. Furthermore, in the tree shrew, the K pathway contains the only pair of laminae that has differential outputs to the striate cortex, suggestive of distinct circuitries: K L3 and K L6 primarily terminate in different subdivisions of Layer 3, with L3 axons targeting 3b and L6 projecting to 3c (Usrey et al., 1992). Finally, previous work has shown that different collicular cell types selectively target K laminae of the tree shrew dLGN, with stellate cells selectively terminating in K L3 while NFV-like cells preferentially target K L6 (Diamond et al., 1991). While our study does not provide definitive clarification for where each tectal cell type projects to, it provides evidence for potential differences in neurotransmitter release from these distinct cell types, thus suggesting differences in excitation and modulation impinging on the two K laminae.

In thalamic circuitry, inputs have been canonically classified as either “drivers” or “modulators” (Sherman and Guillery, 1998, 2002), with retinal input constituting the primary driver across all visual pathways. However, in the mouse, Bickford and colleagues have found that optogenetic stimulation of TG terminals led to excitatory postsynaptic potentials similar to those elicited by optic nerve stimulations, leading them to propose that TG inputs had the signature characteristics of an additional driver input on geniculate W-type relay cells (Bickford et al., 2015). Drivers and modulators are also distinguished by their transporters in the LGN: the driver, typically retinal, input utilizes VGluT2 while cortical input utilizes VGluT1 (Land et al., 2004; Balaram et al., 2011, 2013, 2015; Rovó et al., 2012). The LGN's third glutamatergic input, the one from SC, was speculated to be VGluT2-positive based on morphological and physiological properties of the SC terminals (Bickford et al., 2015) and the presence of VGluT2 mRNA transcript in superficial SC (Balaram et al., 2015), where LGN-projecting stellate cells reside. The current study adds to this evidence by confirming the presence of VGluT2 in SC terminals and VGluT2 mRNA in LGN-projecting stellate cells, which is consistent with the idea that at least some of the TG axons may be exerting driver-like influences on relay cells in K laminae. However, we also demonstrated that not all LGN-projecting cells utilize VGluT2: a substantial portion of geniculate-projecting tectal neurons does not express VGluT2 mRNA, including a morphological subtype of NFV cells, raising the question of which neurotransmitters may be released from those axons. The presence of a nonglutamatergic NFV cell type is not novel: unlike the glutamatergic NFV cells projecting to deep SC (Gale and Murphy, 2014), a subpopulation of NFV cells are GABAergic and project to PBG (Whyland et al., 2020). It will be interesting to know whether those same cells also project to geniculate K laminae via axon collaterals. Similarly, while marginal cells have been shown to be direction-selective (Mooney et al., 1985) and project to the LGN (Mooney et al., 1988), the neurotransmitters expressed in this cell type have not been identified, except for our RNA-FISH experiments, which reveal that they do not express VGluT2.

Our study admittedly has a technical limitation in quantifying the ratio of axons that originate from three different TG cell types that terminate in two K laminae: the tracer injections in the SC are bound to be incomplete in filling all TG cells located across the entire depth and topography of the SC. Thus, the proportion of filled terminal boutons after any injection may represent a greater percentage of one cell type over another. We expect this question could be best addressed with multiple injections throughout the mediolateral extent of the sSC using cell type-specific AAVs, which could allow for the assessment of the prevalence of such axons separately in LGN laminae. Regardless, our findings suggest that not only are the K laminae circuitry heterogeneous but also that two K laminae may be influenced differentially by distinct tectal pathways with unique functions.

### Implications for blindsight

Blindsight, the phenomenon where humans and nonhuman primates can detect visual stimuli following damage to V1 (Sanders et al., 1974), is a condition that must rely on an existing processing stream that relays visual information beyond the striate cortex. There are several features of the K pathway that make it suitable for this purpose. First, tectal projections are a distinct, specific property of the K pathway in all mammalian species examined (Harting et al., 1991; Lachica and Casagrande, 1993; Bickford et al., 2015; Baldwin and Bourne, 2020), and they may exert strong excitation on relay cells, akin to retinal input. Second, SC is the origin of two main parallel projections that bypass V1: one reaches multiple areas of the association cortex through the visual pulvinar, and another to the extrastriate visual cortex via K layers of LGN (Benevento and Yoshida, 1981; Yoshida and Benevento, 1981; Yukie and Iwai, 1981; Bullier and Kennedy, 1983; Rodman et al., 2001). Third, studies in macaques with V1 ablation have shown that while a widespread relay cell degeneration predominate, many cells that express CALB and project to visual association areas survive (Cowey and Stoerig, 1989; Rodman et al., 2001; Atapour et al., 2021, 2022) and maintain intact visual processing (Yu et al., 2018). Similarly, in a monkey model of blindsight, pharmacological inactivation of LGN neurons suppressed functional activation of the visual association cortex and eliminated visual detection abilities (Schmid et al., 2010), suggesting that the geniculate K pathway is crucial for blindsight. Finally, the prominent tectal input we demonstrate in the tree shrew dLGN may be indicative of a specialized visual channel that relays information about stimulus motion and eye movement to higher visual areas. While direct evidence for tree shrew K laminae relay cells projecting to MT analog extrastriate cortical areas in a similar fashion demonstrated in the macaque and marmosets (Sincich et al., 2004; Atapour et al., 2022) is elusive, whether or not distinct K pathway information processed in geniculate L3 and L6 have selective roles in blindsight will be an interesting question to address.

## Conclusion

In a seminal 1994 review (Casagrande, 1994), Vivian Casagrande provided extensive anatomical, neurochemical, and functional evidence for a third parallel visual pathway, the K pathway, suggesting it may play both a modulatory role in vision and a perceptual role during eye movements. By examining tree shrew LGN where K pathways are uniquely segregated, we revealed structural distinctions of parallel pathways and revealed further evidence for two K subpathways, each with their own inputs and distinct synaptic circuitries, and this is consistent with multiple roles K relay cells may play on visual processing as suggested by Casagrande.

## Synthesis

Reviewing Editor: Mark Laubach, American University Decisions are customarily a result of the Reviewing Editor and the peer reviewers coming together and discussing their recommendations until a consensus is reached. When revisions are invited, a fact-based synthesis statement explaining their decision and outlining what is needed to prepare a revision will be listed below. The following reviewer(s) agreed to reveal their identity: Nafiseh Atapour.

Thank you for sending your paper to eNeuro!

It was reviewed by two experts. Their comments are below. Please revise your paper to address all points that were raised.

Please submit Figure 8 (as suggested by one of the reviewers) or a modified version of it for the visual abstract.

Reviewer 1

In this manuscript, the authors characterized cellular properties, the morphological and connectivity properties of synaptic inputs in each of the six layers in the tree-shrew dLGN by combining immunochemistry, electron microscopy (EM), and virus and dye injection. The study provides exciting and important new insights into the tree shrew dLGN from multiple perspectives, particularly regarding the understudied koniocellular (K) layers. The findings reveal that Calbindin+ cells predominantly label relay cells in K layers and that K layers receive distinct types of retinogeniculate inputs from other layers and between the two K layers. Moreover, tectogeniculate terminals specifically target K layers and exhibit retinal terminal-like characteristics. These results suggest that the two K laminae may constitute two distinct pathways with unique functions. Interestingly, tectogeniculate terminals are not co-stained with either VGluT2 or VGluT1, indicating that Lamina 6 receives an additional VGluT2-positive input, distinct from both retinal and tectal inputs. This surprising finding contrasts with previous rodent studies.

I support the publication of these results, but I think the manuscript needs to address a few points for clarification:

Major comments:

1. Line 189-191: 'parvalbumin, ..., surprisingly stained all neuronal cell bodies in the tree shrew reliably demarcating the soma membranes (Fig. 1b) in all geniculate laminae'. Does 'all neuronal cell bodies' refer to every neuron in the dLGN? Since NeuroTrace is not specific to neurons and PV only stained a subset of NeuroTrace+ cells, how did the author confirm that PV stained 'all neuronal cell bodies'?

2. The study used NeuroTrace (NT) and GABA signals to distinguish interneurons from relay cells. However, since NT is not specific to neurons, a subset of the NT+ and GABA- cells could be glial cells. If PV staining can be used as a reliable marker for neurons, why didn't the authors use PV and GABA staining to identify relay cells and interneurons? Related to this, in Line 199-200: 'M/P L5 has the largest soma size on average (301.6 {plus minus} 11.41 μm2) significantly different from every other lamina except K L6 (Fig. 1e, p<.0001).' However, Fig. 1h shows that the mean soma sizes in L4 and L5 are both smaller than 300 um^2, and L4 has the largest mean soma size. Is this discrepancy caused by the different neuronal markers used (PV+ in Fig. 1e and NT+ in Fig. 1h)?

3. The average value of LM terminals in Fig. 4d looks higher than in Fig. 5e, especially for L3. Could the authors perform some statistical tests to examine whether the difference is significant? If it is indeed significant, does that indicate that there is a third type of input that provides large LM terminals beyond RG and TG, or that only a small subset of RG and TG are labeled by dye injection? If it is the latter, how should readers interpret the conclusion in Fig. 3 that RG terminals in L3 are significantly smaller than in other layers, particularly when the LM result in L1, L3, and L6 did not differ significantly?

4. It is important to clarify whether TG terminals contain dark mitochondria, or light mitochondria, or both. Line 289: 'suggesting that there may be another origin of input contributing to the LM population in Koniocellular L3'-this seems to imply that the authors think some of the tectogeniculate terminals contain LM profiles, but they are uncertain since they cannot tell whether labeled TG terminals contain light or dark mitochondria due to DAB chromogen. The manuscript should consistently reflect this uncertainty, for example, when they refer to unlabeled terminals with RLP profiles as retinal terminals. Additionally, how did the authors address the caveat of labeling geniculate branches of retinotectal axons from SC BDA injections (Datskovskaia et al., 2001) in their analysis?

5. The finding that TG terminals in L3 and L6 are neither VGluT2 nor VGluT1 positive is surprising, given rodent studies demonstrated that the majority TG terminals are glutamatergic and that TG terminals in L3 and L6 closely resemble RG terminals.

a. Is it possible to inject retrograde tracers into the dLGN and perform immunostaining or RNA-Fish in the superior colliculus to confirm that the dLGN-projecting SC neurons are not VGluT2 or VGluT1 positive?

b. The TG axons in the dLGN appear to spread beyond L3 ( Fig. 7f). Could the authors outline the dLGN layers in the bottom panel of Fig. 7f to clarify whether there are TG axon terminals in L4? Could the authors also show images around the injection site to illustrate the spread of the virus around the SC?

c. Are the terminals in the pulvinar positive for VGluT2 and/or VGluT1?

Minor comments:

1. Line 232: 'suggesting that interneurons in all laminae express calbindin' could be deleted.

2. Line 249: Could examples of terminals with dark mitochondria that exhibit different sizes across layers be displayed in supplementary figures? Also, explicitly state that retinogeniculate axon terminals have light mitochondria.

3. Line 250: L1-2: the mean is 0.065, whereas the means for other layers are an order of magnitude larger. Is 0.065 correct (rather than 0.65)?

4. Line 268: "besides" should be "except"?

5. Line 267: p < 0.001-.0001. Both ** and *** are displayed in Fig. 3g, suggesting a range of 0.01 to 0.001. The same question for Line 268.

6. The difference between L1 and L6 is not obvious from the histogram presented in Fig. 1b. Could a different data presentation format be used here to better highlight this difference?

7. In Fig. 3c, "non-RG" could be misleading, as it suggests "non-retinogeniculate." Consider labeling them as "unlabeled."

8. In Fig. 3: "RLP: unlabeled retinal terminal with light mitochondria"-light mitochondria can also be in tectogeniculate terminals. Consider removing "retinal."

9. Line 806: "non-RG" should be "non-TG" or "unlabeled".

Reviewer 2

Overall this work shows neurochemistry and connectivity of LGN in Tree shrew and includes detailed analysis on cell size and bouton size/types of retinal and tectal inputs. My detailed comments are below. Some English editing is required.

Line 49: For better readability, the two sentences to be combined

Line 51-52; "laminae 1, 2, 4, and 5 all receive mixed projections comprised of both magnocellular and parvocellular input while laminae 3 and 6 are solely comprised of koniocellular input". Word input better to be replaced with cell or sentence better to be revised as these LGN layers have M/P cells that receive inputs....

Line 64: Sc to be defined

Line 71; "axonal inputs" better than "input axons"

Line 72: better "classified" to be change to "identified"

Line 97: what was these coordinates based on? Any reference?

Line 193: NT as abbreviation to be introduced as used later on.

Line 201-202; This sentence is unclear, and not necessarily correct. The difference in cell size alone does not explain or correlate the morphological distinctions in RGCs.

Line 205-210; NT+ and GABA_ can be glial cell. Also, NT+ and GABA+ cells can be glial cells. In the dataset of 1,504 cell somas (663 relay cells and 841 interneurons) percentage of interneurons is much higher than expected, >55%. This analysis is wrong and has to be taken out of the paper. The required staining for such analysis would be with a neuronal marker such as NeuN not Nissl stain. Authors themselves have pointed out that Nissl stains glia and neurons. Colocalisation with NeuN and GABA can show actunal number of interneurons. Such high percentage of interneurons are against observations in other species, either primates (https://doi.org/10.1007/s00429-021-02257-0) or non-primates (https://doi.org/10.1101/2023.08.10.549394)

Line 214: not a clear sentence

Line 217: a difference is soma size is not indicator of distinct population. The sentence must be rewritten to reflect this

Line 223-4: add references such as https://doi.org/10.1017/s0952523898154044 orhttps://doi.org/10.1146/annurev.neuro.23.1.127. Literature has to be better cited

Line229: "the vast majority of CALB+ somata fully colocalize with GABA", suggests that GABA is also present in relay cells, thus confirming point raised earlier that the percentage of GABA" interneurons seems too high. This is rather an unusual finding that needs further testing to make sure antibody is acting specifically. Different from normal observations in other species, for example presence of GABA in relay cells of LGN after injury (https://doi.org/10.1007/s00429-021-02257-0).

Overall criteria for identifications of cells as interneuros or relay cells better to be clarified.

Line 246-247. "are" to be replaced with "were"

Line 402; Goodchild and Martin 1998 show selective expression of Calb and PV in marmoset LGN not the opposite

## Author Response

Dear Dr. Laubach, We are grateful for the careful and constructive input from the reviewers, and we truly appreciate the opportunity to revise our manuscript. We hope we were able to address all concerns in this revised version of our manuscript.

Below, we provide a detailed, line-by-line response to the reviewers' comments along with the corresponding revisions made. For ease of reading, the reviewers' comments are in Arial and indented, and the authors comments are in Times Roman. Substantial additions in the text are further copied in brackets and highlights.

Synthesis of Reviews:

Computational Neuroscience Model Code Accessibility Comments for Author (Required):N/A Synthesis Statement for Author (Required):

Both reviewers found that the paper was much improved by the revisions. Some concerns remain. The new reviews are given in full below. Please revise the address all points that were raised. Thank you for sending your paper to eNeuro.

Reviewer 1 The revised manuscript shows significant improvement compared to the original submission. I am especially appreciative that the authors conducted two additional experiments- immunostaining of neurotransmitter markers in tectogeniculate axons and RNA-FISH of retrogradely labeled tectogeniculate neurons-to demonstrate the co-expression of VGlut2 in tectogeniculate neurons. Through retrograde tracing (new Figure 8), they identified three distinct types of tectogeniculate neurons and showed that different proportions of these neurons co-expressed VGlut2 mRNA. Findings reported in the manuscript offer valuable insights into the cellular and synaptic morphological and molecular characteristics of LGN neurons from distinct layers and pathways. These insights will also inform future studies to identify potential functional and projection differences among the distinct koniocellular pathways in the tree shrew visual system.

Before recommending the manuscript for publication, I suggest the authors add one more analysis (point 1) to reconcile results from the two new sets of experiments and clarify the analysis in point 2. Additionally, there are some quick analyses that could be performed to support or refute the authors' hypotheses mentioned in the text. Some additional text editing is also needed to improve clarity.

1. VGluT2 co-expression: • The authors stated, 'We found no colocalization of VGluT1 or GABA with anterogradely transported BDA in TG boutons (not shown) in either Lamina 3 or Lamina 6. However, VGluT2-ir was colocalized with BDA in both Koniocellular Lamina 3 and 6 (Figure 7i-o).' (Line 441 and 443).

On the other hand, they noted, 'RNA-FISH labeling revealed that VGluT2 transcript was most prominent in stellate cells, with 88.7% of these cells expressing VGluT2 (Fig. 8h). In contrast, only 16.7% of NFV-like cells and none of the marginal cells expressed VGluT2 mRNA (n = 36 and 30, respectively).' (Line 464-466). The comparison of the two results raises two important questions: 1. What proportions of BDA+ TG boutons colocalize with VGluT2 in Lamina 3 and Lamina 6, respectively; (...) For addressing the proportions of BDA+ TG boutons that colocalize with VGluT2 in Lamina 3 and Lamina 6 (question 1), we were also tempted by bringing a quick answer using the confocal material. However, we refrained from it due to non-feasibility of this analysis using current data. The main problem is admittedly the shortcomings of the immunofluorescence colocalization approaches applied for fiber colocalization questions such as ours: The first shortcoming is that, while tracer molecules fill the entire axon, vesicular protein VGluT2 is only concentrated in the terminal boutons. This means, a 'Tracer Only' region indicates either no colocalization in a terminal bouton (true negative), or an axon shaft where no colocalization could ever be expected in the first place (false negative). The false negative signal can be accounted for by using baseline measurements from a region where no true negative is expected, an approach similar to what we have done for assessing VGluT2 colocalization in retinal terminals in geniculate M/P laminae. We were able to run such analysis for the quantification of retinogeniculate terminals containing VGluT2 immunolabel analysis in our study (Figure 7f). In the analysis illustrated in Figure 7f, we addressed the possibility of false negative scoring in two ways: First we limited our analysis to regions away from white matter or where fiber tracks are expected. As this would not eliminate the tracer filled fibers without boutons coursing through the ROI, we have also devised an approach whereby we normalized the % ratio of retinal label in VGluT2 objects (where true negatives are expected) to the % ratio of VGluT2 label in boutons with retinal signal (where no true negatives can be expected since all retinal boutons express VGluT2) in every region analyzed. This normalization approach eliminated the rate of false positives from both % ratios, and revealed that the rate of normalized colocalization in M/P laminae were around 1, while under 1 in K laminae 3 and 6, and these two groups were significantly different from one another. This graph also reveals that there was more singly-labeled VGluT2 boutons in L6 (L3: 0.70 {plus minus} 0.06 vs. L6: 0.60 {plus minus} 0.05), suggesting differences between 2 K laminae, however, these two laminae are not significantly different from one another (Welch's, p=0.25). The significance bars, which we realized were missing from Figure 7f, are now added, and the text reflects the within K and M/P analyses: "The M/P laminae exhibited indices close to 1 (1.06 {plus minus} 0.03), while the K laminae showed lower values (0.65 {plus minus} 0.04; Fig. 7f), and these two groups were significantly different from one another (Welch's t-test, p<.0001). While there were more singly-labeled VGluT2 boutons in L6 (L3: 0.70 {plus minus} 0.06 vs. L6: 0.60 {plus minus} 0.05), indicating differences between 2 K laminae, a statistical difference was not evident (Welch's, p=0.25)." Lines 458-462 Regarding whether we can apply the same analytical approach to tectogeniculate plus VGluT2 analysis, the answer is, unfortunately, no. Unlike the retinogeniculate axon visualizations, the tectogeniculate visualizations do not present an internal control as a baseline for false negative labeling. Because tectogeniculate axons only project to L3 and L6, and there are no known SC projections that are expected to be 100% VGluT2+, a reliable and proper quantitative analysis of TG and VGluT2 colocalization is not feasible. The confocal analysis of VGluT2 and SC labels gives us the qualitative evidence that VGluT2 is indeed present in SC axons in both L3 and L6. However, the current material is not suitable to obtain background normalization in two laminae, and thus, to assess the false negative colocalization rates. This clarification of this limitation is now added in the results section of the manuscript: "Quantitative assessment for the rate of VGluT2/SC axon colocalization was not feasible, because, unlike for the assessment of VGluT2/Retinal axon described above (Fig. 7f), a background normalization condition, where 100% colocalization could be expected, was not present in SC injection experiments given that axons only project to Koniocellular laminae." Lines 470-474 Further confirmation of the ratios of VGlu2+ and VGluT2- TG axons in L3 vs. L6 will require a more direct approach that is not confounded by the limitations of confocal analyses. Such analysis should also take into account the possibility that VGluT2+ or negative TG axons from STE, NFV, or MAR LGN- projecting cells may not have exclusive selectivity for L3 or L6. The most definitive approach to answer this question would be visualization of SC cell-type specific axons using cell-type specific AAVs, and reassessing the prevalence and colocalization indices for such axons separately in LGN laminae. Our lab has been working on designing such cell type-specific AAVs, and so far we have had success isolating at least one cell-type in the tree shrew SC. We hope to be able to directly address the diversity of SC cell projections in LGN in coming years. However, we now have to acknowledge these caveats as a shortcoming of our study and that further analysis needs to be left outside of the current study. We have now done so and included this as a future study in the Discussion (lines 636- 643). "Our study admittedly has a technical limitation in quantifying the ratio of axons that originate from three different tectogeniculate cell types that terminate in two K laminae: the tracer injections in the SC are bound to be incomplete in filling all tectogeniculate cells located across the entire depth and topography of the SC. Thus, the proportion of filled terminal boutons after any injection may represent a greater percentage of one cell type over another. We expect this question could be best addressed with multiple injections throughout the mediolateral extent of the sSC using cell type-specific AAVs, which could allow for the assessment of the prevalence of such axons separately in LGN laminae." (..) 2. What other neurotransmitters or glutamate transporters are expressed in the VGluT2 mRNA- TG neurons. • Please quantify the percentage of VGluT2+ terminals that are labeled by BDA, and the percentage of BDA+ terminals that are VGluT2+. If all BDA+ TG boutons are VGluT2+, the authors should explain why a substantial portion of retrogradely labeled NFV-like and marginal cells are VGluT2 negative.

As discussed above, it is not feasible to measure the proportion of VGluT2-containing tectogeniculate boutons, nor can we assert that all TG axons were VGluT2+ in any K laminae. As a result, we could only conclude qualitatively that VGluT2 is present at least in some TG terminals. On the other hand, our RNA FISH experiments clearly demonstrated that while majority of TG cells contained VGluT2 mRNA transcript, many TG cells with NFV morphology and all cells with MAR morphology did not, bringing about the possibility that some TG axons may not be glutamatergic. In line with this, it is highly likely that VGluT2 negative NFV cells are GABAergic: Whyland, Slusarczyk, and Bickford (2020) demonstrated that PBG-projecting GABAergic neurons in the SGS displayed narrow field morphologies. On the other hand, we have not encountered any mention of the neurotransmitter content of MAR cells in the literature. But we anticipate that the TG-MAR cell axons may not be represented in our SC injection cases: it is highly likely that MAR cells that are localized right under pia mater in the zonal layer were not filled with our SC injections that typically centered at the middle of the superficial SC. Regardless, the lack of GABA immunostaining in TG+ axons is puzzling (see image of GABA and VG1 immunostaining and tracer filled SC axons, inserted below). We can only surmise that this may be a factor of variable selectivity of anterograde tracer injections for TG cell types. A non-uniform representation of VGluT2+ and VGluT2- TG cells across the SC topography is also conceivable. Further, more detailed analyses with multiple injections throughout the mediolateral extent of the SC would be needed to begin addressing this question. While the possibility of GABAergic transmission from TG axons is ultimately very interesting, we cannot assess the percentage of boutons containing a particular neurotransmitter with the methodology used in this study. Similarly, RNAfish confirmation of GABA transcript in retrogradely filled cells as our original retrograde LGN injection experiment focused on confirming the VGluT2 transcript only and did not include GABA probes. We have now included these in the Discussion. "However, we also demonstrated that not all LGN-projecting cells utilize VGluT2: a substantial portion of geniculate-projecting tectal neurons do not express VGluT2 mRNA, including a morphological subtype of narrow field vertical cells, raising the question of which neurotransmitters may be released from those axons. The presence of a non-glutamatergic NFV cell type is not novel: unlike the glutamatergic NFV cells projecting to deep SC (Gale and Murphy, 2014), a subpopulation of NFV cells are GABAergic and project to PBG (Whyland et al., 2020). It will be interesting to know whether those same cells also project to geniculate K-laminae via axon collaterals. Similarly, while marginal cells have been shown to be direction-selective (Mooney et al., 1985) and project to the LGN (Mooney et al., 1988), the neurotransmitters expressed in this cell type have not been identified, except for our RNA-FISH experiments, which reveal that they do not express VGluT2." (Lines 625-635) • Additionally, please compare these results with the colocalization rate of retinogeniculate terminals with VGluT2 (Figure 6f). Together, the proportions of TG+ VGluT2 terminals and RG+ VGluT2 terminals should not exceed 100%.

Even if we were able to find a baseline brain region for tectogeniculate fibers and run a normalized colocalization analysis similar to our retinotectal fiber analysis, we would argue that numbers from RG and TG experiments would essentially be incomparable due to different tracer transport and immunopenetration ratios that are invariably expected in two separate tissue samples. Even though eye injections may reasonably fill all RGC axons, tracer injections to SC are always incomplete, that is, confined to the injection site, which is only a small percentage of the SC topography. We are as frustrated as this reviewer that the questions regarding the molecular specificity of three tectogeniculate cell types and their projection selectivity in two geniculate K laminae cannot be resolved with the anatomical approaches used in our study. We acknowledge this as a shortcoming of our study in the discussion (lines 627-643), and respectfully refrain from adding an inconclusive analysis. We hope the reviewer agrees that our study provides novel evidence toward the complexity of tectogeniculate regulation and warrants detailed examination of this question using more conclusive cell-type specific anatomical and functional tools.

Lastly, the authors should also mention in the discussion that the negative results of RNA-FISH may also be caused by the sensitivity of the detection method, unless the authors have a positive control showing all the VGluT2+ cells could be detected by the VGluT2 probe in RNAFISH.

We are indeed confident about the specificity and selectivity of the RNA fish visualization for VGluT2.

The strongest positive control we can offer is the pattern of VGluT2 in the lower SGS, where VGluT2+ Wide Field Vertical cells reside. A thick band of cell bodies positive for VGluT2 transcript is seen in the lower SGS and upper SO (see image below), essentially serving as a positive control for our detection method. VGluT2 transcript is also strongly present in upper SGS where STE and NFV cells reside. In addition, retrogradely filled STE and NFV cells in upper SGS showed very different colocalization percentages- a result that could not be explained by a low sensitivity of the VGluT2 probe for SC cells in general.

2. Cell sampling strategy: • In the rebuttal letter, the authors clarified, 'In fact, to be able to compare the soma sizes (not the prevalence) of relay vs interneurons, our experimental design aimed to sample at least 80- 100 relay cells and 80-100 interneurons from each lamina. As a result, our dataset with 1,504 somas (now with 1,592, since we sampled more cells for the analyses in this revision to ensure each lamina surpassed 100 cells per type) aimed to have a representative sample of both relay cells and interneurons and is not intended to reflect the true proportion of each in LGN.' This helpful information needs to be added around line 274 ('In a dataset of 1,592 cell somas (714 relay cells and 878 interneurons; > 100 cells per lamina'). Otherwise, readers may mistakenly think that 714 and 878 reflect the ratio of relay cells and interneurons in the LGN, as unbiased sampling is typically used in the literature.

Thank you for this suggestion. We have added this clarification to the text: "As relay cells are encountered more frequently than interneurons in the LGN, we employed a sampling strategy that aimed to collect at least 100 cells of each type from each lamina, thereby oversampling interneurons. Thus, the dataset is optimized to compare size differences across cell types and laminae, but it is not suitable for revealing the ratio of relay vs. interneuron density in any given region." Lines 282- 286. • Related to this sampling issue, in Figure 2m, the authors show the proportion of CALB+ cells that are relay cells versus interneurons in each lamina. It seems that the proportions of relay cells and interneurons add together to 100% for each lamina, implying that the majority of CALB1+ cells in L1-L5 are interneurons; and the majority of CALB1+ cells in L3 and L6 are relay cells. However, it is unclear whether the cells are sampled unbiasedly or biasedly sampled by using the same strategy as for sampling cells to quantify cell sizes. Please state the sampling strategy clearly in the text. The unbiased method should be used here.

Yes, the reviewer's interpretation is correct. Our sampling strategy by design was unbiased toward evaluating every calbindin-stained cells encountered in a given region, followed by assessing GABA stain in each of those identified CALB cells. This strategy unavoidably yielded different numbers of CALB cells encountered in each lamina, but allowed us to assess calbindin's selectivity for GABA- vs. GABA- cells in M/P vs K laminae. We have now clarified our methodology in the text to emphasize that every CALB+ cell was assessed for GABA colocalization across multiple tissue sections, and thus Figure 2m shows the proportion of each cell type that makes up the total CALB+ population in all analyzed cells. "To elucidate differences in CALB+ relay cells and interneurons in these distinct pathways, the tissue was dually stained, CALB+ cells were sampled across all laminae in seven LGN tissues sections (n=1539), and the colocalization of GABA was assessed in those cells (Fig. 2a-b, g-l). K laminae displayed the majority of CALB+ cells (L3: 794, L6: 232), and ~20% of those contained GABA, suggesting that calbindin is utilized not only in relay cells but also in interneurons. In contrast, while CALB+ cells in M/P laminae were not as numerous (50-190 cells), 65-95% of CALB+ somata contained GABA (Fig. 2g-i, m), suggesting that calbindin is indeed expressed in the M/P laminae, and preferentially in interneurons (Fig.

2m). It should be noted that as these experiments did not include imaging of a third channel for all geniculate cells, they do not reveal whether all K relay cells, or all M/P interneurons, are calbindin- positive. Regardless, they provide definitive confirmation that interneurons across all laminae contain calbindin, however only relay neurons in K-laminae predominantly express this calcium binding protein." Lines 308-320 3. Line 266-268: The statement 'K laminae are also composed of two distinct cell types, potentially revealing differences between L3 and L6.' can be readily tested by plotting the histogram of soma sizes of L3 and L6 neurons separately in Figure 1g. On the other hand, soma sizes in L3 and L6 are not significantly different as shown in Figure 1i and 1j, suggesting that this hypothesis may not hold.

We thank the reviewer for highlighting this - it prompted us to plot the NT+ cell size distributions for K- L3 and K-L6 separately, which revealed very similar distributions, consistent with the relay cell analyses displayed in Fig. 1i and 1j. Similarly, we are not able to differentiate if ON/OFF or ipsi/contra pairs of M/P laminae contain any of the M/P three relay cell subpopulations selectively. Given this, we have altered the text to better represent what our findings may suggest for the Koniocellular relay population: "Third, K laminae are also composed of two distinct cell types. Cell size distributions between K-L3 and K-L6 show similar trends and do not significantly differ, so this finding may support the existence of multiple K relay cell subtypes." Lines 274-277 4. Line 329: Integrate the result stated in the text here into Fig 3c? We thank the reviewer for this suggestion. These results have now been added to Fig. 3c as its own inset, and we have added statistics to emphasize this result. The text and figure legends have been corrected on lines 353-356 and 968-971, respectively.

5. Line 325-331: What about moving the text from 'In all laminae' in Line 325 to the end of 331 to be after the middle of line 336? (..) The text has been moved to the suggested position (lines 350-352). (..)Moreover, the average unlabeled terminals with dark mitochondria in K layers is 0.88 +/- 0.06 [i)m2, whereas the average TG terminals in L3 has 1.41+/- 0.09 [i)m2, in L6 has 1.69 +/- 0.10 [i)m2. Please provide explanation for the significant differences in the average terminal sizes from the two measurements.

The population of unlabeled terminals with dark mitochondria contain multiple populations in addition to TG input. These other terminal populations (from cortex, TRN, interneurons, and brainstem) typically constitute small to medium-sized terminals in geniculate laminae. Thus, the entire dark mitochondria population, made up of terminals from multiple origins, should not be directly compared to the terminal cross-section areas of labeled TG terminals. The other populations certainly account for the significant differences in average terminal sizes.

6. Line 359-360: The result 'M/P-recipient L1 contains a greater percentage of large-sized terminals' is not directly reflected in the histogram, especially between L1 and L6. Could the authors add another dashed line at a larger value of terminal area and label the percentage on top. In principle, the percentage of L1 boutons with a value smaller than the highlighted larger value should be smaller than the percentage for L3 and L6.

We thank the reviewer for bringing this to our attention. In addition to showing the proportion of terminals smaller than 0.2um2, we have now calculated the proportion of terminals greater than 1.5um2 and added a dashed line on all three histograms in Figure 4b to highlight the differences. The changes are reflected in Figure 4b and figure legend (lines 980-982).

7. Line 372-373: Another possibility is that tracers did not label all retinogeniculate terminals.

We agree, all tracer injections come with a probability factor for incomplete filling. This factor can be very large for injections placed in large structures such as superior colliculus or cortical areas. The eye injections may also be subjected to this error factor, as a result of potential incomplete spread of the tracer in the vitreous. In our experiments, the projection pattern of retinogeniculate terminals, that is, the presence of fibers in all relevant laminae and for all receptive field topography, indicated that the tracer placed in the vitreous spread sufficiently to reach all RGCs representing the entire topographic field. That is why we are more confident that the efficacy of RGC fillings were close to 100%, with the proviso that we cannot control for the differential efficacy of tracer transport in different axons. The possibility of variable axonal transport in different RGC types is now acknowledged in the text (lines 335-338). Regardless, the presence of large terminals in L3-6 with or without light mitochondria criterion is consistent with previous and current findings suggesting selective presence of tectogeniculate axons in K- laminae, and that measurable morphological similarities exist between retinal and tectal terminals. In stating "This suggests that either retinal terminals do not account for all terminals with light mitochondria or that mitochondrial appearance is not a reliable criterion for retinal terminal identification, at least in Koniocellular L3" (lines 372-373), we meant to provide a refinement of RLP morphological criteria that are commonly used in ultrastructural studies of the LGN.

8. Line 387: 'synaptic connections', 'connections' is indicating their connections to target neurons. However, only synaptic morphological characteristics were examined here. Please modify.

Thank you. This is now clarified in the text: "We examined morphological properties of a total of 526 synaptic terminals" Line 410 9. Figure 6c: When a TG terminal makes triad connections, it will synapse both on F2 and relay cell dendrites. In this scenario, will both 'dendrites' and F2 terminals be counted as 'postsynaptic targets'? If so, wouldn't the percentages of dendrites and F2 sum to more than 100%? Please clarify.

Thank you for asking for clarification. The math, in this case, is correct. If a given terminal is making more than one synapse, the postsynaptic target for each synapse is counted and recorded as a relay dendrite or the dendritic appendage of an interneuron (F2 profile). As a result, the number of synapses in the dataset is equal to the number of postsynaptic targets, not the number of terminals. Because the proportion of synapses targeting F2s vs dendrites is out of the total number of synapses, the number of synapses is equal to the number of relay and F2 postsynaptic targets. That is, the ratio of relay dendrite targets + the ratio of F2 targets = 100.

10. Line 8: please confirm the usage of 'selectivity' here.

We regret the typo. This is now corrected as 'selectively'.

11. Line 15: The use of 'only' is inaccurate, since Calbindin is also present in interneurons.

The abstract has been edited to clarify this statement and accurately represent the results of calbindin expression: "While confirming that K-laminae relay cells contain calbindin, our study also found calbindin expression in GABAergic cells across all laminae." Lines 12-14 12. Line 16: Add a period between 'targets' and 'VGluT2'.

Thank you. This is corrected.

13. Line 469: The statement 'but only one appears to provide...' is inaccurate.

Corrected as 'but only one appears to provide most of the VGluT2-positive input'. Line 499 Reviewer 2 This new version is improved. All my comments have been addressed except the staining by NT (Nissl staining). It is not clear to me why a simple NeuN staining (neuron specific marker) has not been done to resolve the controversy. At least it could have been done in a case for the results to be better interpreted. The least authors must mention these as shortcoming of the study.

We have now addressed advantages and limitations of choosing a Nissl stain over neuron-specific markers like NeuN (lines 245-251). "For measuring soma sizes, we weighed the advantages and limitations of a histochemical stain Neurotrace (NT; Thermofisher) and the antibody based NeuN staining to visualize geniculate cells. While selective for neuronal nuclear protein, NeuN stain inherits limitations including dependence on antibody penetration and potential exclusion of some neuron types in the brain (Kumar and Buckmaster, 2007). On the other hand, Neurotrace reliably labels all cells with Nissl bodies, including glial cells (García- Cabezas et al., 2016). For our purposes, NT proved preferable because glial cell somas can be excluded based on size criteria." 1) Some glial cells are GABAergic, for example see this review doi: 10.3389/fncel.2022.892497, therefore a cell that is NT+ and GABA+ can also be a glial cell.

We agree that this is an important consideration in choosing the marker for neuron soma size quantifications. We have acknowledged this possibility and cited the suggested review (lines 245-256).

2) Some of glial cell bodies are larger than the interneurons, so deciding cell type based on size is far from ideal. There would be many glial cells that have been included in analysis in this study as they can be larger than the cut off of 40 μm2. This is my estimation based on other species.

We were unable to confirm the assertion that glial somata could be larger than 40 um2. In comprehensive studies that assessed the morphological properties of glial cells, including astrocytes and oligodendrocytes, the average size of glia were estimated to be 4-7 micron in diameter (<30 um2) (Stolzenburg et al., 1989; Rajkowska et al., 1989) or 25um2 (Verdonk et al., 2016) in various brain regions and species. While hypertrophied glial cells are encountered in neurodegenerative conditions including AD, stroke and accelerated aging, (Savage JC, Carrier M, Tremblay ME, 2019; doi:

10.1007/978-1-4939-9658-2_2.), the microglia are generally recognized for their somata smaller then 50um2 (Jinno S, Fleischer F, Eckel S et al., 2007; doi: 10.1002/glia.20552). We encountered only one study that reported microglia cell body perimeter measurements yielding soma sizes as large as 90um2 (Kongsui et al., 2014 https://doi.org/10.1186/s12974-014-0182-7; Table 1 reports cell body perimeter= ~35um, area= ~90um2), however the examination of methods suggests that this measure refers to perimeter of entire microglia including ramifications. As a result, we are confident that 40um2 cutoff criteria we used effectively eliminated most if not all glial cells from neuronal soma measurement datasets relying on Nissl markers. "...To that aim, we measured the sizes of geniculate GABA+ somata (n=878; >100 cells per laminae, 125.2 {plus minus} 1.3 μm2; Fig. 1h) and determined that 99% of GABAergic cells were larger than 40 μm2, which is larger than glia measured in various species (Stolzenburg et al., 1989; Rajkowska et al., 1998; Verdonk et al., 2016; Liu et al., 2022). Any NT cells smaller than this cutoff value were classified as GABA+ glial cells and excluded from soma size analyses." For the Visual Abstract: I would have liked a more simplified visual abstract. Some concepts are hard to grasp from this current visual abstract and requires a lot of attention and comparison between the parts.

We apologize for our misunderstanding. The visual abstract has now been redesigned as a more simplified version. We hope these edits help to demonstrate the main findings with better clarity.

## References

[B1] Albano JE, Norton TT, Hall WC (1979) Laminar origin of projections from the superficial layers of the superior colliculus in the tree shrew, *Tupaia glis*. Brain Res 173:1–11. 10.1016/0006-8993(79)91090-4 90538

[B2] Arya S, Mount DM, Netanyahu NS, Silverman R, Wu AY (1998) An optimal algorithm for approximate nearest neighbor searching fixed dimensions. J ACM 45:891–923. 10.1145/293347.293348

[B3] Atapour N, Worthy KH, Rosa MGP (2021) Neurochemical changes in the primate lateral geniculate nucleus following lesions of striate cortex in infancy and adulthood: implications for residual vision and blindsight. Brain Struct Funct 226:2763–2775. 10.1007/s00429-021-02257-0 33743077

[B4] Atapour N, Worthy KH, Rosa MGP (2022) Remodeling of lateral geniculate nucleus projections to extrastriate area MT following long-term lesions of striate cortex. Proc Natl Acad Sci U S A 119:e2117137119. 10.1073/pnas.2117137119 35058366 PMC8794847

[B5] Balaram P, Takahata T, Kaas JH (2011) VGLUT2 mRNA and protein expression in the visual thalamus and midbrain of prosimian galagos (*Otolemur garnettii*). Eye Brain 3:5–15. 10.2147/EB.S16998 22984342 PMC3442197

[B6] Balaram P, Hackett TA, Kaas JH (2013) Differential expression of vesicular glutamate transporters 1 and 2 may identify distinct modes of glutamatergic transmission in the macaque visual system. J Chem Neuroanat 50–51:21–38. 10.1016/j.jchemneu.2013.02.007 23524295 PMC3695749

[B7] Balaram P, Isaamullah M, Petry HM, Bickford ME, Kaas JH (2015) Distributions of vesicular glutamate transporters 1 and 2 in the visual system of tree shrews (*Tupaia belangeri*). J Comp Neurol 523:1792–1808. 10.1002/cne.23727 25521420 PMC4470886

[B8] Baldwin MKL, Bourne JA (2020) The evolution of subcortical pathways to the extrastriate cortex. In: *Evolutionary neuroscience* (Kaas JH, ed), pp 565–587. Cambridge, MA: Academic Press.

[B9] Benevento LA, Yoshida K (1981) The afferent and efferent organization of the lateral geniculo-prestriate pathways in the macaque monkey. J Comp Neurol 203:455–474. 10.1002/cne.902030309 6274921

[B10] Benson DL, Isackson PJ, Hendry SH, Jones EG (1991) Differential gene expression for glutamic acid decarboxylase and type II calcium-calmodulin-dependent protein kinase in basal ganglia, thalamus, and hypothalamus of the monkey. J Neurosci 11:1540–1564. 10.1523/JNEUROSCI.11-06-01540.1991 1646294 PMC6575400

[B11] Bickford ME, Slusarczyk A, Dilger EK, Krahe TE, Kucuk C, Guido W (2010) Synaptic development of the mouse dorsal lateral geniculate nucleus. J Comp Neurol 518:622–635. 10.1002/cne.22223 20034053 PMC4278806

[B12] Bickford ME, Zhou N, Krahe TE, Govindaiah G, Guido W (2015) Retinal and tectal “driver-like” inputs converge in the shell of the mouse dorsal lateral geniculate nucleus. J Neurosci 35:10523–10534. 10.1523/JNEUROSCI.3375-14.2015 26203147 PMC4510292

[B13] Blatow M, Caputi A, Burnashev N, Monyer H, Rozov A (2003) Ca2+ buffer saturation underlies paired pulse facilitation in calbindin-D28k-containing terminals. Neuron 38:79–88. 10.1016/S0896-6273(03)00196-X 12691666

[B14] Bullier J, Kennedy H (1983) Projection of the lateral geniculate nucleus onto cortical area V2 in the macaque monkey. Exp Brain Res 53:168–172. 10.1007/BF00239409 6201379

[B15] Callaway EM (2005) Structure and function of parallel pathways in the primate early visual system. J Physiol 566:13–19. 10.1113/jphysiol.2005.088047 15905213 PMC1464718

[B16] Campbell CBG, Jane JA, Yashon D (1967) The retinal projections of the tree shrew and hedgehog. Brain Res 5:406–418. 10.1016/0006-8993(67)90047-9 6035943

[B17] Casagrande VA (1994) A third parallel visual pathway to primate area V1. Trends Neurosci 17:305–310. 10.1016/0166-2236(94)90065-5 7524217

[B18] Catterall WA, Few AP (2008) Calcium channel regulation and presynaptic plasticity. Neuron 59:882–901. 10.1016/j.neuron.2008.09.005 18817729

[B19] Chen B, Hu X-J, Pourcho RG (1996) Morphological diversity in terminals of W-type retinal ganglion cells at projection sites in cat brain. Vis Neurosci 13:449–460. 10.1017/S0952523800008129 8782372

[B20] Chen C, Blitz DM, Regehr WG (2002) Contributions of receptor desensitization and saturation to plasticity at the retinogeniculate synapse. Neuron 33:779–788. 10.1016/S0896-6273(02)00611-6 11879654

[B21] Conley M, Fitzpatrick D, Diamond IT (1984) The laminar organization of the lateral geniculate body and the striate cortex in the tree shrew (*Tupaia glis*). J Neurosci 4:171–197. 10.1523/JNEUROSCI.04-01-00171.1984 6198492 PMC6564764

[B22] Conway JL, Schiller PH (1983) Laminar organization of tree shrew dorsal lateral geniculate nucleus. J Neurophysiol 50:1330–1342. 10.1152/jn.1983.50.6.1330 6663330

[B23] Cowey A, Stoerig P (1989) Projection patterns of surviving neurons in the dorsal lateral geniculate nucleus following discrete lesions of striate cortex: implications for residual vision. Exp Brain Res 75:631–638. 10.1007/BF00249914 2744120

[B24] Dacey DM (2000) Parallel pathways for spectral coding in primate retina. Annu Rev Neurosci 23:743–775. 10.1146/annurev.neuro.23.1.743 10845080

[B25] Dacey DM, Packer OS (2003) Colour coding in the primate retina: diverse cell types and cone-specific circuitry. Curr Opin Neurobiol 13:421–427. 10.1016/S0959-4388(03)00103-X 12965288

[B26] Datskovskaia A, Carden WB, Bickford ME (2001) Y retinal terminals contact interneurons in the cat dorsal lateral geniculate nucleus. J Comp Neurol 430:85–100. 10.1002/1096-9861(20010129)430:1<85::AID-CNE1016>3.0.CO;2-K 11135247

[B27] DeBruyn EJ 3rd (1983) The organization and central terminations of retinal ganglion cells in the tree shrew (Tupaia glis). Vanderbilt University.

[B28] Diamond IT, Conley M, Fitzpatrick D, Raczkowski D (1991) Evidence for separate pathways within the tecto-geniculate projection in the tree shrew. Proc Natl Acad Sci U S A 88:1315–1319. 10.1073/pnas.88.4.1315 1705034 PMC51008

[B29] Diamond IT, Fitzpatrick D, Schmechel D (1993) Calcium binding proteins distinguish large and small cells of the ventral posterior and lateral geniculate nuclei of the prosimian galago and the tree shrew (*Tupaia belangeri*). Proc Natl Acad Sci U S A 90:1425–1429. 10.1073/pnas.90.4.1425 8434002 PMC45886

[B30] Drenhaus U, Von Gunten A, Rager G (1997) Classes of axons and their distribution in the optic nerve of the tree shrew (*Tupaia belangeri*). Anat Rec 249:103–116. 10.1002/(SICI)1097-0185(199709)249:1<103::AID-AR13>3.0.CO;2-T 9294655

[B31] Eiber CD, Rahman AS, Pietersen ANJ, Zeater N, Dreher B, Solomon SG, Martin PR (2018) Receptive field properties of koniocellular on/off neurons in the lateral geniculate nucleus of marmoset monkeys. J Neurosci 38:10384–10398. 10.1523/JNEUROSCI.1679-18.2018 30327419 PMC6596204

[B32] Enroth-Cugell C, Robson JG (1966) The contrast sensitivity of retinal ganglion cells of the cat. J Physiol 187:517–552. 10.1113/jphysiol.1966.sp008107 16783910 PMC1395960

[B33] Erişir A, Van Horn SC, Sherman SM (1997) Relative numbers of cortical and brainstem inputs to the lateral geniculate nucleus. Proc Natl Acad Sci U S A 94:1517–1520. 10.1073/pnas.94.4.1517 9037085 PMC19823

[B34] Erişir A, Van Horn SC, Sherman SM (1998) Distribution of synapses in the lateral geniculate nucleus of the cat: differences between laminae a and A1 and between relay cells and interneurons. J Comp Neurol 390:247–255. 10.1002/(SICI)1096-9861(19980112)390:2<247::AID-CNE7>3.0.CO;2-1 9453668

[B35] Fitzpatrick D, Carey RG, Diamond IT (1980) The projection of the superior colliculus upon the lateral geniculate body in *Tupaia glis* and *Galago senegalensis*. Brain Res 194:494–499. 10.1016/0006-8993(80)91230-5 7388626

[B36] Friedlander MJ, Lin CS, Stanford LR, Sherman SM (1981) Morphology of functionally identified neurons in lateral geniculate nucleus of the cat. J Neurophysiol 46:80–129. 10.1152/jn.1981.46.1.80 7264710

[B37] Gale SD, Murphy GJ (2014) Distinct representation and distribution of visual information by specific cell types in mouse superficial superior colliculus. J Neurosci 34:13458–13471. 10.1523/JNEUROSCI.2768-14.2014 25274823 PMC4180477

[B38] García-Cabezas MÁ, John YJ, Barbas H, Zikopoulos B (2016) Distinction of neurons, glia and endothelial cells in the cerebral cortex: an algorithm based on cytological features. Front Neuroanat 10:107. 10.3389/fnana.2016.00107 27847469 PMC5088408

[B39] Godwin DW, Horn SCV, Erişir A, Sesma M, Romano C, Sherman SM (1996) Ultrastructural localization suggests that retinal and cortical inputs access different metabotropic glutamate receptors in the lateral geniculate nucleus. J Neurosci 16:8181–8192. 10.1523/JNEUROSCI.16-24-08181.1996 8987843 PMC6579199

[B40] Goodchild AK, Martin PR (1998) The distribution of calcium-binding proteins in the lateral geniculate nucleus and visual cortex of a new world monkey, the marmoset, *Callithrix jacchus*. Vis Neurosci 15:625–642. 10.1017/S0952523898154044 9682866

[B41] Graham J, Casagrande VA (1980) A light microscopic and electron microscopic study of the superficial layers of the superior colliculus of the tree shrew (*Tupaia glis*). J Comp Neurol 191:133–151. 10.1002/cne.901910108 7400390

[B42] Guillery RW (1969) The organization of synaptic interconnections in the laminae of the dorsal lateral geniculate nucleus of the cat. Z Für Zellforsch Mikrosk Anat 96:1–38. 10.1007/BF00321474 5772028

[B43] Harting JK, Huerta MF, Hashikawa T, van Lieshout DP (1991) Projection of the mammalian superior colliculus upon the dorsal lateral geniculate nucleus: organization of tectogeniculate pathways in nineteen species. J Comp Neurol 304:275–306. 10.1002/cne.903040210 1707899

[B44] Hasse JM, Bragg EM, Murphy AJ, Briggs F (2019) Morphological heterogeneity among corticogeniculate neurons in ferrets: quantification and comparison with a previous report in macaque monkeys. J Comp Neurol 527:546–557. 10.1002/cne.24451 29664120 PMC6192868

[B45] Hendry SHC, Jones EG (1991) GABA neuronal subpopulations in cat primary auditory cortex: co-localization with calcium binding proteins. Brain Res 543:45–55. 10.1016/0006-8993(91)91046-4 2054675

[B46] Hendry SHC, Reid RC (2000) The koniocellular pathway in primate vision. Annu Rev Neurosci 23:127–153. 10.1146/annurev.neuro.23.1.127 10845061

[B47] Hendry SHC, Yoshioka T (1994) A neurochemically distinct third channel in the macaque dorsal lateral geniculate nucleus. Science 264:575–577. 10.1126/science.8160015 8160015

[B48] Holdefer RN, Norton TT (1995) Laminar organization of receptive field properties in the dorsal lateral geniculate nucleus of the tree shrew (*Tupaiaglis belangeri*). J Comp Neurol 358:401–413. 10.1002/cne.903580307 7560294

[B49] Irvin GE, Norton TT, Sesma MA, Casagrande VA (1986) W-like response properties of interlaminar zone cells in the lateral geniculate nucleus of a primate (*Galago crassicaudatus*). Brain Res 362:254–270. 10.1016/0006-8993(86)90450-6 3942875

[B50] Jayakumar J, Roy S, Dreher B, Martin PR, Vidyasagar TR (2013) Multiple pathways carry signals from short-wavelength-sensitive (‘blue’) cones to the middle temporal area of the macaque. J Physiol 591:339–352. 10.1113/jphysiol.2012.241117 23070701 PMC3630789

[B51] Johnson JK, Casagrande VA (1995) Distribution of calcium-binding proteins within the parallel visual pathways of a primate (*Galago crassicaudatus*). J Comp Neurol 356:238–260. 10.1002/cne.903560208 7629317

[B52] Jones EG, Hendry SHC (1989) Differential calcium binding protein immunoreactivity distinguishes classes of relay neurons in monkey thalamic nuclei. Eur J Neurosci 1:222–246. 10.1111/j.1460-9568.1989.tb00791.x 12106154

[B53] Kaas JH, Huerta MF, Weber JT, Harting JK (1978) Patterns of retinal terminations and laminar organization of the lateral geniculate nucleus of primates. J Comp Neurol 182:517–553. 10.1002/cne.901820308 102662

[B54] Kaplan E (2003) The M, P, and K pathways of the primate visual system. Vis Neurosci 1:481–493. 10.7551/mitpress/7131.001.0001

[B55] Kaplan E (2014) The M, P and K pathways of the primate visual system revisited. In: *The new visual neurosciences* (Werner JS, Chalupa LS, eds), pp 215–226. Cambridge, MA: MIT Press.

[B56] Kerschensteiner D, Guido W (2017) Organization of the dorsal lateral geniculate nucleus in the mouse. Vis Neurosci 34:E008. 10.1017/S0952523817000062 28965501 PMC6380502

[B57] Kumar SS, Buckmaster PS (2007) Neuron-specific nuclear antigen NeuN is not detectable in gerbil substantia nigra pars reticulata. Brain Res 1142:54–60. 10.1016/j.brainres.2007.01.027 17291468 PMC2691720

[B58] Lachica EA, Casagrande VA (1993) The morphology of collicular and retinal axons ending on small relay (W-like) cells of the primate lateral geniculate nucleus. Vis Neurosci 10:403–418. 10.1017/S0952523800004648 8494795

[B59] Land PW, Kyonka E, Shamalla-Hannah L (2004) Vesicular glutamate transporters in the lateral geniculate nucleus: expression of VGLUT2 by retinal terminals. Brain Res 996:251–254. 10.1016/j.brainres.2003.10.032 14697503

[B60] LeVay S, Ferster D (1977) Relay cell classes in the lateral geniculate nucleus of the cat and the effects of visual deprivation. J Comp Neurol 172:563–584. 10.1002/cne.901720402 190274

[B61] Liu J, Feng X, Wang Y, Xia X, Zheng JC (2022) Astrocytes: GABAceptive and GABAergic cells in the brain. Front Cell Neurosci 16:892497. 10.3389/fncel.2022.892497 35755777 PMC9231434

[B62] Lu HD, Petry HM (2003) Temporal modulation sensitivity of tree shrew retinal ganglion cells. Vis Neurosci 20:363–372. 10.1017/S0952523803204028 14658765

[B63] Maher EE, Briegel AC, Imtiaz S, Fox MA, Golino H, Erisir A (2023) 3D electron microscopy and volume-based bouton sorting reveal the selectivity of inputs onto geniculate relay cell and interneuron dendrite segments. Front Neuroanat 17:1150747. 10.3389/fnana.2023.1150747 37007643 PMC10064015

[B64] Mooney RD, Klein BG, Rhoades RW (1985) Correlations between the structural and functional characteristics of neurons in the superficial laminae and the hamster’s superior colliculus. J Neurosci 5:2989–3009. 10.1523/JNEUROSCI.05-11-02989.1985 4056863 PMC6565159

[B65] Mooney RD, Nikoletseas MM, Ruiz SA, Rhoades RW (1988) Receptive-field properties and morphological characteristics of the superior collicular neurons that project to the lateral posterior and dorsal lateral geniculate nuclei in the hamster. J Neurophysiol 59:1333–1351. 10.1152/jn.1988.59.5.1333 3385463

[B66] Norton TT, Casagrande VA, Irvin GE, Sesma MA, Petry HM (1988) Contrast-sensitivity functions of W-, X-, and Y-like relay cells in the lateral geniculate nucleus of bush baby, *Galago crassicaudatus*. J Neurophysiol 59:1639–1656. 10.1152/jn.1988.59.6.1639 3404199

[B67] Norton TT, Casagrande VA (1982) Laminar organization of receptive-field properties in lateral geniculate nucleus of bush baby (*Galago crassicaudatus*). J Neurophysiol 47:715–741. 10.1152/jn.1982.47.4.715 6279794

[B68] Rajkowska G, Selemon LD, Goldman-Rakic PS (1998) Neuronal and glial somal size in the prefrontal cortex: a postmortem morphometric study of schizophrenia and Huntington disease. Arch Gen Psychiatry 55:215–224. 10.1001/archpsyc.55.3.215 9510215

[B69] Rodieck RW, Watanabe M (1993) Survey of the morphology of macaque retinal ganglion cells that project to the pretectum, superior colliculus, and parvicellular laminae of the lateral geniculate nucleus. J Comp Neurol 338:289–303. 10.1002/cne.903380211 8308173

[B70] Rodman HR, Sorenson KM, Shim AJ, Hexter DP (2001) Calbindin immunoreactivity in the geniculo-extrastriate system of the macaque: implications for heterogeneity in the koniocellular pathway and recovery from cortical damage. J Comp Neurol 431:168–181. 10.1002/1096-9861(20010305)431:2<168::AID-CNE1063>3.0.CO;2-N 11169998

[B71] Rovó Z, Ulbert I, Acsády L (2012) Drivers of the primate thalamus. J Neurosci 32:17894–17908. 10.1523/JNEUROSCI.2815-12.2012 23223308 PMC3672843

[B72] Roy S, Jayakumar J, Martin PR, Dreher B, Saalmann YB, Hu D, Vidyasagar TR (2009) Segregation of short-wavelength-sensitive (S) cone signals in the macaque dorsal lateral geniculate nucleus. Eur J Neurosci 30:1517–1526. 10.1111/j.1460-9568.2009.06939.x 19821840 PMC2777259

[B73] Sanders MD, Warrington EK, Marshall J, Wieskrantz L (1974) “Blindsight”: vision in a field defect. Lancet 1:707–708. 10.1016/S0140-6736(74)92907-9 4132425

[B74] Schmid MC, Mrowka SW, Turchi J, Saunders RC, Wilke M, Peters AJ, Ye FQ, Leopold DA (2010) Blindsight depends on the lateral geniculate nucleus. Nature 466:373–377. 10.1038/nature09179 20574422 PMC2904843

[B75] Scrucca L, Fop M, Murphy TB, Raftery AE (2016) Mclust 5: clustering, classification and density estimation using Gaussian finite mixture models. R J 8:289. 10.32614/RJ-2016-021 27818791 PMC5096736

[B76] Sherman SM, Guillery RW (1998) On the actions that one nerve cell can have on another: distinguishing “drivers” from “modulators”. Proc Natl Acad Sci U S A 95:7121–7126. 10.1073/pnas.95.12.7121 9618549 PMC22761

[B77] Sherman SM, Guillery RW (2002) The role of the thalamus in the flow of information to the cortex. Philos Trans R Soc Lond B Biol Sci 357:1695–1708. 10.1098/rstb.2002.1161 12626004 PMC1693087

[B78] Sincich LC, Park KF, Wohlgemuth MJ, Horton JC (2004) Bypassing V1: a direct geniculate input to area MT. Nat Neurosci 7:1123–1128. 10.1038/nn1318 15378066

[B79] Solomon SG, White AJR, Martin PR (2002) Extraclassical receptive field properties of parvocellular, magnocellular, and koniocellular cells in the primate lateral geniculate nucleus. J Neurosci 22:338–349. 10.1523/JNEUROSCI.22-01-00338.2002 11756517 PMC6757604

[B80] Stanford LR, Friedlander MJ, Sherman SM (1981) Morphology of physiologically identified W-cells in the C laminae of the cat’s lateral geniculate nucleus. J Neurosci 1:578–584. 10.1523/JNEUROSCI.01-06-00578.1981 7346569 PMC6564179

[B81] Stanford LR (1987) W-cells in the cat retina: correlated morphological and physiological evidence for two distinct classes. J Neurophysiol 57:218–244. 10.1152/jn.1987.57.1.218 3549992

[B82] Stolzenburg J-U, Reichenbach A, Neumann M (1989) Size and density of glial and neuronal cells within the cerebral neocortex of various insectivorian species. Glia 2:78–84. 10.1002/glia.440020203 2524445

[B83] Tailby C, Solomon SG, Peirce JW, Metha AB (2007) Two expressions of “surround suppression” in V1 that arise independent of cortical mechanisms of suppression. Vis Neurosci 24:99–109. 10.1017/S0952523807070022 17430613

[B84] Tamamaki N, Uhlrich DJ, Sherman SM (1995) Morphology of physiologically identified retinal X and Y axons in the cat’s thalamus and midbrain as revealed by intraaxonal injection of biocytin. J Comp Neurol 354:583–607. 10.1002/cne.903540408 7608339

[B85] Usrey WM, Muly EC, Fitzpatrick D (1992) Lateral geniculate projections to the superficial layers of visual cortex in the tree shrew. J Comp Neurol 319:159–171. 10.1002/cne.903190113 1375607

[B86] Van Hooser SD, Heimel JAF, Nelson SB (2003) Receptive field properties and laminar organization of lateral geniculate nucleus in the gray squirrel (*Sciurus carolinensis*). J Neurophysiol 90:3398–3418. 10.1152/jn.00474.2003 12840084

[B87] Van Horn SC, Erişir A, Sherman SM (2000) Relative distribution of synapses in the A-laminae of the lateral geniculate nucleus of the cat. J Comp Neurol 416:509–520. 10.1002/(SICI)1096-9861(20000124)416:4<509::AID-CNE7>3.0.CO;2-H 10660881

[B88] Verdonk F, et al. (2016) Phenotypic clustering: a novel method for microglial morphology analysis. J Neuroinflammation 13:153. 10.1186/s12974-016-0614-7 27317566 PMC4912769

[B89] Watakabe A, Ohtsuka M, Kinoshita M, Takaji M, Isa K, Mizukami H, Ozawa K, Isa T, Yamamori T (2015) Comparative analyses of adeno-associated viral vector serotypes 1, 2, 5, 8 and 9 in marmoset, mouse and macaque cerebral cortex. Neurosci Res 93:144–157. 10.1016/j.neures.2014.09.002 25240284

[B90] Westerink RHS, Beekwilder JP, Wadman WJ (2012) Differential alterations of synaptic plasticity in dentate gyrus and CA1 hippocampal area of Calbindin-D28K knockout mice. Brain Res 1450:1–10. 10.1016/j.brainres.2012.02.036 22405690

[B91] White AJR, Wilder HD, Goodchild AK, Sefton AJ, Martin PR (1998) Segregation of receptive field properties in the lateral geniculate nucleus of a new-world monkey, the marmoset *Callithrix jacchus*. J Neurophysiol 80:2063–2076. 10.1152/jn.1998.80.4.2063 9772261

[B92] White AJR, Solomon SG, Martin PR (2001) Spatial properties of koniocellular cells in the lateral geniculate nucleus of the marmoset *Callithrix jacchus*. J Physiol 533:519–535. 10.1111/j.1469-7793.2001.0519a.x 11389209 PMC2278639

[B93] Whyland KL, Slusarczyk AS, Bickford ME (2020) GABAergic cell types in the superficial layers of the mouse superior colliculus. J Comp Neurol 528:308–320. 10.1002/cne.24754 31396959 PMC6888991

[B94] Xu X, Ichida JM, Allison JD, Boyd JD, Bonds AB, Casagrande VA (2001) A comparison of koniocellular, magnocellular and parvocellular receptive field properties in the lateral geniculate nucleus of the owl monkey (*Aotus trivirgatus*). J Physiol 531:203–218. 10.1111/j.1469-7793.2001.0203j.x 11179404 PMC2278453

[B95] Yoshida K, Benevento LA (1981) The projection from the dorsal lateral geniculate nucleus of the thalamus to extrastriate visual association cortex in the macaque monkey. Neurosci Lett 22:103–108. 10.1016/0304-3940(81)90071-9 6164960

[B96] Yu H-H, Atapour N, Chaplin TA, Worthy KH, Rosa MG (2018) Robust visual responses and normal retinotopy in primate lateral geniculate nucleus following long-term lesions of striate cortex. J Neurosci 38:3955–3970. 10.1523/JNEUROSCI.0188-18.2018 29555856 PMC6705928

[B97] Yukie M, Iwai E (1981) Direct projection from the dorsal lateral geniculate nucleus to the prestriate cortex in macaque monkeys. J Comp Neurol 201:81–97. 10.1002/cne.902010107 7276252

[B98] Zhou J-N, Ni R-J (2016) *The tree shrew (Tupaia belangeri chinensis) brain in stereotaxic coordinates*. Singapore: Springer.

